# Circulating miRNAs as potential biomarkers of therapy effectiveness in rheumatoid arthritis patients treated with anti-TNFα

**DOI:** 10.1186/s13075-015-0555-z

**Published:** 2015-03-09

**Authors:** Carmen Castro-Villegas, Carlos Pérez-Sánchez, Alejandro Escudero, Ileana Filipescu, Miriam Verdu, Patricia Ruiz-Limón, Ma Angeles Aguirre, Yolanda Jiménez-Gomez, Pilar Font, Antonio Rodriguez-Ariza, Juan Ramon Peinado, Eduardo Collantes-Estévez, Rocío González-Conejero, Constantino Martinez, Nuria Barbarroja, Chary López-Pedrera

**Affiliations:** Maimonides Institute for Research in Biomedicine of Cordoba (IMIBIC)/Reina Sofia University Hospital/University of Cordoba, Avenida Menendez Pidal S-N, E-14004 Cordoba, Spain; Iuliu Hatieganu University of Medicine and Pharmacy, Str. Emil Isac Nr. 13, 400023 Cluj-Napoca, Romania; Department of Medical Sciences, Faculty of Medicine of Ciudad Real, University of Castilla-La Mancha, Calle Altagracia, 50, 13003 Cuidad Real, Spain; Regional Centre for Blood Donation, University of Murcia, IMIB-Arrixaca, Campus Espinardo, E-30100 Murcia, Spain

## Abstract

**Introduction:**

The advent of anti-tumor necrosis factor alpha (anti-TNFα) drugs has considerably improved medical management in rheumatoid arthritis (RA) patients, although it has been reported to be ineffective in a fraction of them. MicroRNAs (miRNAs) are small, non-coding RNAs that act as fine-tuning regulators of gene expression. Targeting miRNAs by gain or loss of function approaches have brought therapeutic effects in various disease models. The aim of this study was to investigate serum miRNA levels as predictive biomarkers of response to anti-TNFα therapy in RA patients.

**Methods:**

In total, 95 RA patients undergoing anti-TNFα/disease-modifying antirheumatic drugs (anti-TNFα/DMARDs) combined treatments were enrolled. Serum samples were obtained at 0 and 6 months and therapeutic efficacy was assessed. miRNAs were isolated from the serum of 10 patients before and after anti-TNFα/DMARDs combination therapy, cDNA transcribed and pooled, and human serum miRNA polymerase chain reaction (PCR) arrays were performed. Subsequently, selected miRNAs were analyzed in a validation cohort consisting of 85 RA patients. Correlation studies with clinical and serological variables were also performed.

**Results:**

Ninety percent of RA patients responded to anti-TNFα/DMARDs combination therapy according to European League Against Rheumatism (EULAR) criteria. Array analysis showed that 91% of miRNAS were overexpressed and 9% downregulated after therapy. Functional classification revealed a preponderance of target mRNAs involved in reduction of cells maturation - especially on chondrocytes - as well as in immune and inflammatory response, cardiovascular disease, connective tissue and musculoskeletal system. Six out of ten miRNAs selected for validation were found significantly upregulated by anti-TNFα/DMARDs combination therapy (miR-16-5p, miR-23-3p, miR125b-5p, miR-126-3p, miRN-146a-5p, miR-223-3p). Only responder patients showed an increase in those miRNAs after therapy, and paralleled the reduction of TNFα, interleukin (IL)-6, IL-17, rheumatoid factor (RF), and C-reactive protein (CRP). Correlation studies demonstrated associations between validated miRNAs and clinical and inflammatory parameters. Further, we identified a specific plasma miRNA signature (miR-23 and miR-223) that may serve both as predictor and biomarker of response to anti-TNFα/DMARDs combination therapy.

**Conclusions:**

miRNA levels in the serum of RA patients before and after anti-TNFα/DMARDs combination therapy are potential novel biomarkers for predicting and monitoring therapy outcome.

**Electronic supplementary material:**

The online version of this article (doi:10.1186/s13075-015-0555-z) contains supplementary material, which is available to authorized users.

## Introduction

Rheumatoid arthritis (RA) is a systemic, inflammatory, autoimmune disorder of unknown etiology that affects primarily the articular cartilage and bone. Characteristic features of RA pathogenesis are persistent inflammation, synovium hyperplasia and cartilage erosion accompanied by joint swelling and joint destruction [1]. Early treatment can prevent severe disability and lead to remarkable patient benefits, although a lack of therapeutic efficiency in a considerable number of patients remains problematic.

Tumor necrosis factor alpha (TNFα) plays a central role in the pathogenesis of RA and is instrumental in causing joint destruction, the clinical hallmark of the disease. It induces macrophages and other cells to secrete proinflammatory cytokines (that is interleukin (IL)-1, IL-6 and IL-8), leads to T cell activation, and induces endothelial cells to express adhesion molecules [2]. TNFα is involved in the differentiation and maturation of osteoclasts (the main cells involved in arthritic bone destruction), and stimulates fibroblasts, osteoclasts and chondrocytes to release proteinases, which destroy articular cartilage and bone [2,3].

The introduction of anti-TNF therapy has significantly improved the outlook for patients suffering from RA. Yet, a substantial proportion of patients fail to respond to these therapies [4]. Treatment response is likely to be multifactorial; however, variation in genes or their expression may identify those most likely to respond [5]. By targeted testing of variants within candidate genes, potential predictors of anti-TNF response have been reported [6]. However, very few markers have been replicated consistently between studies. Other potential serum biomarkers of response have also been explored including cytokines and autoantibodies, with antibodies developing to the anti-TNF drugs themselves being correlated with treatment failure [7-9].

More recently, epigenetic anomalies are emerging as key pathogenic features of RA. The effects of epigenetics in RA range from contributing to complex disease mechanisms to identifying biomarkers for early diagnosis and response to therapy. Key epigenetic areas in RA have been evaluated namely DNA methylation, histone modification, and expression and/or function of microRNAS [10]. MicroRNAs (miRNAs) are small, non-coding RNAs that, depending upon base pairing to messenger RNA (mRNA) mediate mRNA cleavage, translational repression or mRNA destabilization. miRNAs are involved in crucial cellular processes and their dysregulation has been described in many cell types in different diseases [1]. In fact, abnormalities in miRNA expression related to inflammatory cytokines, T helper 17 (Th-17) and regulatory T cells as well as B cells have been described in several autoimmune diseases [11]. Over the past several years it has become clear that alterations exist in the expression of miRNAs in patients with RA. Increasing number of studies have shown that dysregulation of miRNAs in peripheral blood mononuclear cells [12], isolated T lymphocytes [13], synovial tissue and synovial fibroblasts - that are considered key effectors cells in joint destruction - [14-16], contributes to inflammation, degradation of extracellular matrix and invasive behavior of resident cells. Moreover, altered expression of miRNA in plasma and synovial fluid of RA patients has been demonstrated [17].

It has been established that miRNAs can be aberrantly expressed even in the different stages of RA progression, allowing miRNAs to help monitor disease severity and understand its pathogenesis [10]. Yet, to date no study has evaluated the changes that occurred in the profile of serum miRNAs in RA patients after anti-TNFα therapy. Therefore, to identify possible biomarkers predictive of the therapeutic effect of anti-TNFα drugs in RA, we investigated serum miRNA changes after 6 months of treatment.

## Methods

### Patients

Ninety-five RA patients were included in the study (during a period of 24 months) after obtaining approval from the ethics committee of the Reina Sofia Hospital from Cordoba (Spain). All the RA patients fulfilled the American College of Rheumatology criteria for the classification of RA [18]. Patients provided written informed consent.

All patients had inadequate response to at least two disease-modifying antirheumatic drugs (DMARDs), one of which was methotrexate. Patients received DMARDs in monotherapy or in combination therapy. Only patients who were naïve to anti-TNFα agents were included in the study. Therapy with anti-TNFα agents was stable during the study and was associated to DMARDs (Table S5 in Additional file 1). Within the cohort, 55 patients were given infliximab (IFX; 3 mg/kg/day intravenous infusion at times 0, 2 and 6 weeks, and every 8 weeks thereafter); 25 received etanercept (ETA, 25 mg subcutaneously twice weekly), and 15 patients were treated with adalimumab (ADA; 40 mg subcutaneously every week) for 6 months. Blood samples were obtained before the start and at the end of the treatment. To avoid blood composition changes promoted by diet and circadian rhythms, samples were always collected in the early hours in the morning and after a fasting period of 8 hours.

Clinical and laboratory parameters of the RA patients included in the treatment protocols are displayed in Table 1. Patients were evaluated clinically and analytically at baseline (T1) and 6 months of treatment (T2). Clinical assessment included swollen joint count (SJC), tender joint count (TJC), visual analog scale of pain (VAS; range 1 to 100 mm) of patient and clinician, simple disease activity index (SDAI), health assessment questionnaire (HAQ) and number of DMARDs associated with anti-TNFα treatment. Serological evaluation included analysis of rheumatoid factor (RF), anti-cyclic citrullinated peptide antibodies (anti-CCPs), C-reactive protein (CRP, mg/L) and erythrocyte sedimentation rate (ESR, mm/h).

**Table 1:** **Clinical characteristics of rheumatoid arthritis patients recruited to the study**

	**Exploratory cohort**	**Validation cohort**
	**(n = 10)**	**(n = 85)**
**Sex (male/female)**	1/9	11/74
**Age, mean (range)**	54.6 (38-74)	53.6 (24-72)
**Disease duration (y), mean (range)**	10.1 (2-23)	10.4 (1-36)
**Smoking, number (%)**	4 (40%)	23 (27.1%)
**TJC, mean**	13.7 ± 5.8	15.7 ± 4.6
**SJC, mean**	16.9 ± 6.5	11.5 ± 3.7
**DAS28, mean**	5.9 ± 0.7	5.7 ± 0.6
**SDAI**	39.7 ± 14.9	36.3 ± 10.9
**HAQ**	2.14 ± 0.5	2.1 ± 0.3
**ESR (mm), mean**	55 ± 18.62	55.9 ± 16.6
**CRP (mg/dL), mean**	3.6 ± 1.12	3.8 ± 2.1
**Positive rheumatoid factor, n (%)**	7 (70%)	60 (70.6%)
**Positive anti-CCP antibody, n (%)**	4 (40%)	59 (69.4%)
**Medication, n (%)**		
**Infliximab**	9 (90%)	46 (54.1%)
**Etanercept**	1 (10%)	24 (28.2%)
**Adalimumab**	0	15 (17.6%)
**Corticoids, n(%)**	4 (40%)	55 (64.7%)
**Hydroxychloroquine, n (%)**	3 (30%)	22 (25.9%)
**Azathioprine, n (%)**	0 (0%)	5 (5.9%)
**Metotrexate, n (%)**	7 (70%)	58 (68.2%)
**Sulfasalazine, n (%)**	1 (10%)	7 (8.2%)
**Cyclosporine, n (%)**	1 (10%)	2 (2.4%)
**Leflunomide, n (%)**	2 (20%)	33 (38.8%)

TJC, tender joint count; SJC, swollen joint count; DAS28, disease activity score; SDAI, simplified disease activity index; HAQ, health assessment questionnaire; ESR, erythrocyte sedimentation rate; CRP, C-reactive protein; anti-CCP, anti-cyclic citrullinated peptide antibodies.

Response to anti-TNFα/DMARDs combination treatment was assessed by the (European League Against Rheumatism (EULAR) criteria, based on the 28-joint disease activity score (DAS28). The patients were categorized into responders and non-responders based on the change in the DAS28 score. An improvement in DAS28 over ≥1.2 and a DAS28 value ≤3.2 after 6 months of treatment was considered a good response; a DAS28 value after 6 months between 3.2 and 5.1 and a reduction between 0.6 and 1.2 was considered a moderate response. Both of them were considered responders to the therapy. DAS28 score at T2 > 5.1 or a reduction in DAS28 under 0.6 was considered a non-response.

### Blood sample collection and assessment of biological parameters

Whole blood from subjects was collected by direct venous puncture either, into tubes with ethylenediaminetetraacetic acid (EDTA) as an anticoagulant, or into specific tubes for obtaining serum. All the blood was processed for the isolation of plasma or serum within 4 h of collection. The blood was processed by spinning at 2,000 × g for 10 min at room temperature. Then, plasma and serum were transferred to a fresh RNase-free tube and stored at −80°C. RF was measured by immunoturbidimetric assay (Quantia RF kit, Abbot Laboratories, Chicago, IL, USA) and concentrations >30 IU/mL were considered positive. Determination of anti-CCP antibody was tested with the EDIA™ anti-CCP kit (Euro Diagnostica, Malmö, Sweden). Positive anti-CCP titers were considered at a concentration of >5 U/mL.

Plasmatic levels of IL-6, IL-4, IL-17, IL-22, IL-23, monocyte chemotactic protein (MCP-1), TNFα, soluble TNF receptor II (sTNFRII) and vascular endothelial growth factor (VEGF) at T1 and T2 were quantified using cytofluorometry-based enzyme-linked immunosorbent assay (ELISA) technique in accordance with manufacturer’s instructions using FlowCytomix kit (eBioscience, San Diego, CA, USA). Results were calculated using the FlowCytometry Pro software (eBioscience).

### Isolation of microRNAs from serum

Total RNA, including the miRNA fraction, was extracted from serum by using the QIAzol miRNeasy kit (Qiagen, Valencia, CA, USA) with some modifications. A total of 200 μl of serum were thawed on ice and lysed in 1 mL QIAzol Lysis Reagent (Qiagen). Samples in QIAzol were incubated at room temperature for 5 min to inactivate RNases. To adjust for variations in RNA extraction and/or copurification of inhibitors, 5 fmol of spike-in non-human synthetic miRNA (*C. elegans* miR-39 miRNA mimic: 5′-UCACCGGGUGUAAAUCAGCUUG-3′) were added to the samples after the initial denaturation. The remaining extraction protocol was performed according to the manufacturer’s instruction. Total RNA was eluted in 14 μl of RNase-free water and stored at −80°C.

### MicroRNA expression profiling

To identify the changes that occurred in the expression levels of miRNAs in serum from patients treated with anti-TNFα/DMARDs combination therapy, a Human Serum & Plasma miRNA PCR array (Qiagen) was performed. This array profiles the expression of 84 miRNAs detectable and differentially expressed in serum, plasma, and other bodily fluids. Those miRNAs have been carefully selected based on published results that suggest a correlation with serum expression levels and specific diseases. A pool with 2 μl from RNA purified from 10 optimal responder RA patients before treatment, and another pool with 2 μl from RNA purified from the same 10 patients after treatment was performed.

In a reverse-transcription reaction using miScript HiSpec Buffer from the miScript II RT kit (Qiagen), mature miRNAs were polyadenylated by poly(A) polymerase and subsequently converted into cDNA by reverse transcriptase with oligo-dT priming. The formulation of miScript HiSpec Buffer facilitated the selective conversion of mature miRNAs into cDNA, while the conversion of long RNAs, such as mRNAs was suppressed. As a result, background signals potentially contributed by long RNA were non-existent.

The cDNA prepared in a reverse-transcription reaction was used as a template for real-time PCR analysis using miScript miRNA PCR array (which contains miRNA-specific miScript Primer Assays) and the miScript SYBR Green kit, (which contains the miScript Universal Primer -reverse primer- and QuantiTect SYBR Green PCR Master Mix). To profile the mature miRNA expression, a premix of cDNA, miScript Universal Primer, QuantiTect SYBR Green PCR Master MIX, and RNAse-free water, was added to a miScript miRNA PCR array. That array was provided in a 96-well plate format and included replicates of a miRNA reverse transcription control assay (miRTC) and a positive PCR control (PPC). Those were the quality control assays used to determine the presence of reverse transcription and real-time PCR inhibitors.

Raw data were analyzed with the data analysis software for miScript miRNA PCR arrays. The expression levels of miRNAs were normalized to the mean of spiked-in miRNA Cel-miR-39 and were calculated using the 2^-∆∆Ct^ method.

### Quantitative real-time PCR

A fixed volume of 3 μl of RNA solution from the 14 μl-eluate from RNA isolation of 200 μl serum sample was used as input into the reverse transcription. Input RNA was reverse transcribed using the TaqMan miRNA Reverse Transcription kit and miRNA-specific stem-loop primers (Life Technologies, Madrid, Spain). The reaction was conducted in a GeneAmp PCR System 9700 (Life Technologies) at 16°C for 30 min, 42°C for 30 min and 85°C for 5 min. A preamplification step was performed at 95°C for 10 min, 20 cycles of 95°C for 15 seconds, and 60°C for 4 min. Real-time PCR was carried out on a Roche LightCycler 480 (Roche Applied Science, Penzberg, Germany) at 95°C for 10 min, followed by 40 cycles of 95°C for 15 s and 60°C for 1 min using the TaqMan microRNA assay together with TaqMan Universal PCR Master Mix, No AmpErase UNG (Applied Biosystems, San Francisco, CA, USA). Data were normalized to the mean of spiked-in miRNA Cel-miR-39. The Ct mean values of the spiked-in miRNA Cel-miR-39 in the groups T1 and T2 were 16.10 and 16.20 respectively. BestKeeper software was used to evaluate whether this miRNA was a good reference miRNA [19]. After uploading each Ct value in the Excel spreadsheet, the BestKeeper standard deviation (SD) value was lower than 1, thus considering this miRNA as a good stable housekeeping gene for our experimental conditions. The expression levels of miRNAs were calculated using the 2^-∆∆Ct^ method.

### Statistical analysis

All data were expressed as mean ± SD. Statistical analyses were performed with SSPS 17.0 (SPSS Inc., Chicago, IL, USA). Following normality and equality of variance tests, clinical characteristics were compared using paired Student’s *t* test or alternatively by a nonparametric test (Mann-Whitney rank sum test). Paired samples within the same subjects were compared by Wilcoxon signed-rank test. Differences among groups of treatment were analyzed by repeated measures ANOVA. Correlations were assessed by Spearman’s rank correlation. Differences were considered significant at *P* <0.05.

Receiver operating characteristic (ROC) curve analyses, plotting the true positive rate (sensitivity) vs. the false positive rate (1-specificity) at various threshold settings were performed for serum miRNAs, and the areas under the curve (AUCs) were calculated with SPSS. ROC analysis for miRNA-combined, arithmetic mean of level expression was calculated from two miRNAs selected with the highest efficiency values. *P* values <0.05 were considered statistically significant.

## Results

### Clinical response to anti-TNFα/DMARDs combination therapy

Within the cohort, 83 patients were female (87%) and 12 male (13%) with median disease duration of 9 (4 to 14) years. At the start of the anti-TNFα/DMARDs combination therapy all subjects showed high disease activity, reflected by a mean DAS28 of 5.67 (5.29 to 6.11) despite a median number of 2.35 DMARDS agents used concomitantly (range 1 to 3). All patients took non-steroidal anti-inflammatory drugs (NSAIDs) daily and 65% of them received steroid treatment (range 5 to 15 mg/d prednisone). Methotrexate alone or in combination was administered in 68.4% of subjects.

According to DAS28 response criteria, 90% of patients were responders to anti-TNFα/DMARDs combination therapy. At 6 months of therapy most of the clinical parameters evaluated (including TJC, SJC, SDAI, and HAQ) improved significantly. All three biological agents had a favorable influence on the evolution of those parameters (Tables S1 and S2 in Additional file 1). Several autoimmune and serological parameters (such as RF, PCR, ESR, IL6, IL17, and TNFα) were further significantly reduced when patients were classified in responder vs. non-responder (Table 2). Thus, we chose the time after starting therapy to assess serum miRNAs changes.

**Table 2:** **Changes operated on clinical and laboratory parameters after anti-TNFα/DMARDs treatment in responder and non-responder RA patients**

	**Responders (N = 85)**			**Non-responders (N = 10)**		
	**Before anti-TNF treatment**	**After anti-TNF treatment**	***P***	**Before anti-TNF treatment**	**After anti-TNF treatment**	***P***
Clinical assessments						
TJC	15.9 ± 4.8	5.1 ± 2.2	0.000	15.6 ± 5.1	8.5 ± 2.9	0.005
SJC	11.7 ± 3.9	2.9 ± 1.8	0.000	11.6 ± 4.9	5.1 ± 2.7	0.005
DAS28	5.8 ± 0.6	3.3 ± 0.7	0.000	5.5 ± 0.7	4.3 ± 0.6	0.005
SDAI	36.3 ± 11.4	2.9 ± 6.8	0.000	39.2 ± 11.8	5.7 ± 12.1	0.005
HAQ	2.1 ± 0.3	1 ± 0.4	0.000	2.1 ± 0.3	1 ± 0.3	0.008
Serological assessments						
ESR (mm/h)	56.4 ± 17.2	27.9 ± 18.5	0.000	51.4 ± 11.9	37.6 ± 19.3	ns
CRP (mg/L)	3.9 ± 2.1	1.5 ± 1.2	0.000	2.2 ± 0.8	2.5 ± 0.9	ns
RF (U/L)	155.2 ± 288.3	85.4 ± 241	0.000	61.2 ± 84.3	20.8 ± 39.9	0.027
IL-6 (pg/mL)	9.4 ± 46.7	1.4 ± 6.8	0.000	2.1 ± 5.6	0	ns
TNF (pg/mL)	14.9 ± 28.8	5.7 ± 6.1	0.038	4.1 ± 4.5	4.7 ± 5.1	ns
sTNFRII (pg/mL)	1.5 ± 0.9	1.5 ± 0.7	ns	1.3 ± 0.4	1.5 ± 0.7	ns
MCP-1 (pg/mL)	1174.8 ± 1615.4	1216.4 ± 1655.4	ns	740 ± 504.6	678.6 ± 221.8	ns
VEGF (pg/mL)	1107.7 ± 1360.9	830.3 ± 570.1	ns	796.7 ± 462.7	1077.9 ± 711.6	ns
IL23 (pg/mL)	115 ± 235.5	76.8 ± 132.9	ns	41.2 ± 30.5	223.5 ± 424.8	ns
IL22 (pg/mL)	46.3 ± 112	17.2 ± 29.2	ns	8.4 ± 8.1	104.9 ± 193.3	ns
IL17 (pg/mL)	4.8 ± 8.5	2.1 ± 2.1	0.024	11.9 ± 19.1	1.4 ± 1.4	ns
IL4 (pg/mL)	22.8 ± 17.6	14.9 ± 16.2	ns	4.2 ± 6	12.8 ± 18.2	ns

TNFα, tumor necrosis factor alpha; DMARDS, disease-modifying antirheumatic drugs; RA, rheumatoid arthritis; T1 baseline; T2 at 6 months; TJC, tender joint count; SJC, swollen joint count; DAS28, disease activity score; SDAI, simplified disease activity index; HAQ, health assessment questionnaire; ESR, erythrocyte sedimentation rate; CRP, C-reactive protein; RF, rheumatoid factor; IL-6, interleukin 6; TNF, tumor necrosis factor; sTNFRII, soluble tumor necrosis factor receptor type II; MCP-1, monocyte chemoattractant protein 1; VEGF, vascular endothelial growth factor; IL-23, interleukin 23; IL-22, interleukin 22; IL-17, interleukin 17; IL-4, interleukin 4.

### Differentially expressed miRNAs in the serum of RA patients before and after anti-TNFα/DMARDs combination therapy

**Figure 1:**
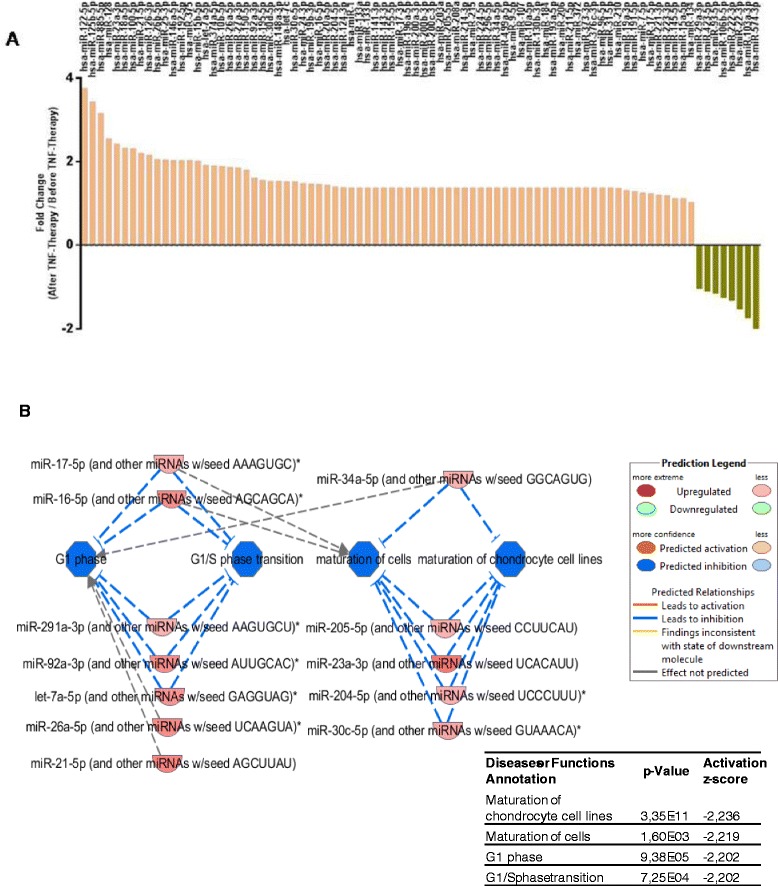
**Plasma miRNA profiling using miRNA array. (A)** To identify the changes that occurred in the expression levels of miRNAs in serum from patients treated with anti-TNFα/DMARDs combination therapy, Human Serum & Plasma miRNA PCR array (Qiagen) was performed. In this profile, the expression levels of 75 miRNAs were found increased, while 9 miRNAs decreased after treatment. **(B)** All the miRNAs modified after anti-TNFα/DMARDs treatment together with the observed fold change were analyzed using the IPA software in order to find interrelationships and potential impact on specific pathways. Among all the targets, the most significant findings (*P* value <0.01) indicated that maturation of cells and transition to G1 phase were inhibited. DMARDs, disease-modifying antirheumatic drugs; IPA, Ingenuity Pathway Analysis; miRNAs, microRNAs; TNFα, tumor necrosis factor alpha.

To evaluate the expression of serum miRNAs before and after anti-TNFα/DMARDs combination therapy, we profiled miRNA spectra from pools of RNA purified from 10 RA serum samples before treatment and 10 RA serum samples after treatment (exploratory cohort). We tested 84 miRNAs during this analysis process. In this profile, the expression levels of 75 miRNAs were found increased, while 9 miRNAs decreased after treatment (Figure 1A).

A detailed analysis of the altered miRNAs in response to anti-TNFα/DMARDs combination treatment, by using the Ingenuity Pathway Analysis (IPA), showed that a large number of them had target mRNAs involved in immune and inflammatory response, cardiovascular system development and function, or connective tissue and musculoskeletal system. Interestingly, among all the potential effects, we found a cluster of miRNAs (which increased after therapy) that may result in a significant impact on the reduction of the maturation of cells, especially on chondrocytes. Also our results seem to indicate that G1 phase transition may be inhibited (Figure 1B).

### Validation of the differentially expressed miRNAs

**Figure 2:**
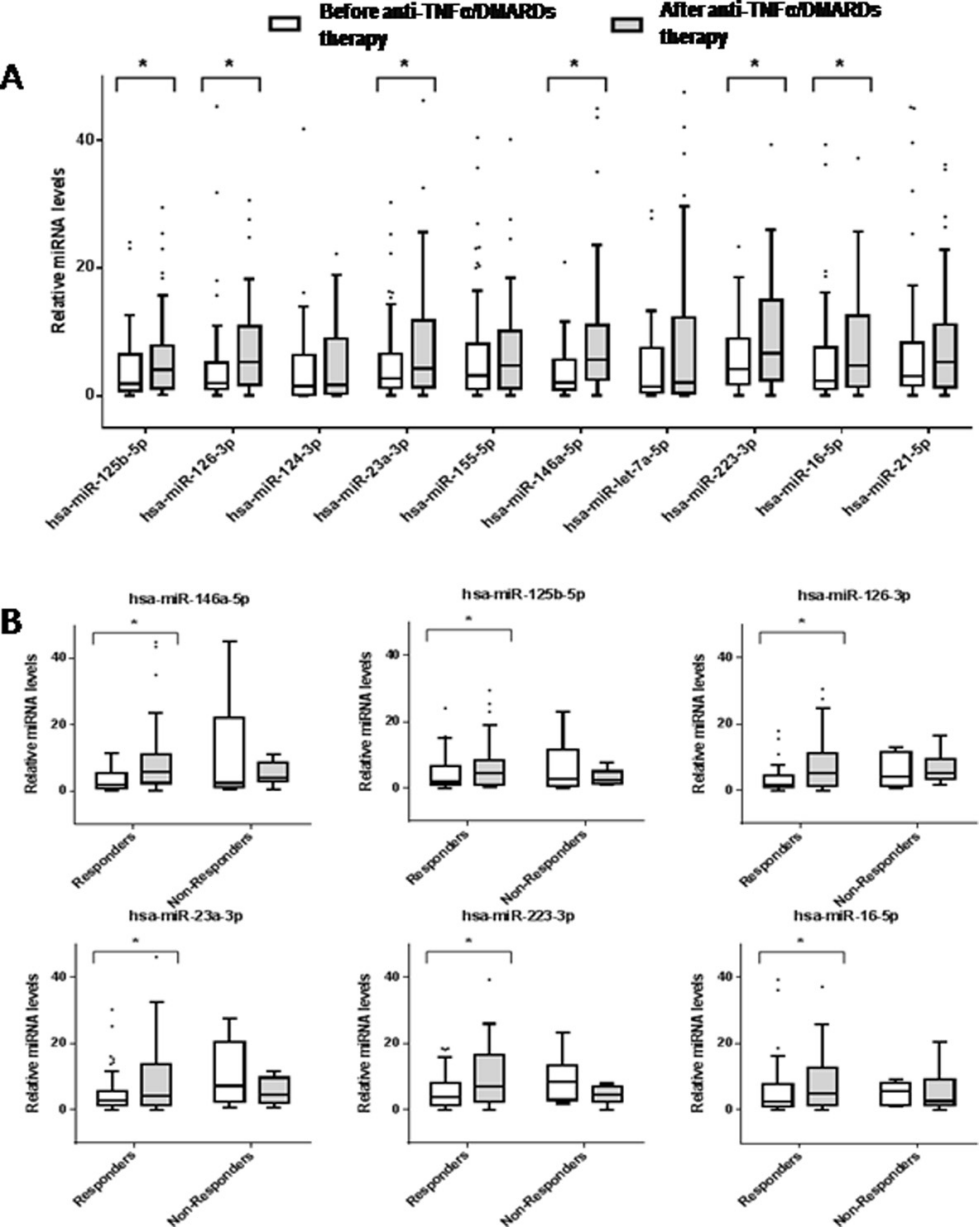
**Relative miRNA levels at start (T1) and after six months of anti-TNFα/DMARDs combination therapy (T2) in the validation cohort (n = 85).** To validate the PCR array data, ten miRNAs differentially expressed were selected (hsa-miR-125b, hsa-miR-23a-3p, hsa-miR-21-5p, hsa-miR-126-3p, hsa-miR-146a-5p, hsa-let-7a-5p, hsa-miR-16-5p, hsa-miR-124a-3p, hsa-miR-155-5p, and hsa-miR-223). **(A)** Relative expression levels of each miRNA are shown. Boxes indicate the interval between the 25^th^ and 75^th^ percentiles and horizontal bars inside boxes indicate median. Whiskers indicate the interval of data within 1.5 × interquartile ranges (IQR). Closed circles indicate data points outside 1.5 x IQR. ^*^ *P* <0.05. **(B)** Comparison of relative change of miRNA levels between responders and non-responders groups for the six miRNAs found significantly altered after therapy in the validation cohort.^*^ *P* <0.05. DMARDs, disease-modifying antirheumatic drugs; miRNAs, microRNAs; TNFα, tumor necrosis factor alpha.

To validate the PCR array data, five miRNAs differentially expressed, showing at least 2-fold change between the two conditions, were selected (hsa-miR-125b, hsa-miR-23a-3p, hsa-miR-21-5p, hsa-miR-126-3p and hsa-miR-146a-5p). A second group of five miRNAs under 2-fold change but involved in processes such as inflammation, cardiovascular and autoimmune diseases, and RA were also selected (hsa-let-7a-5p, hsa-miR-16-5p, hsa-miR-124a-3p, hsa-miR-155-5p, hsa-miR-223-3p). The changes that occurred in the expression of the selected miRNAs were evaluated in all the patients included in the study. In total population, six of the ten miRNAs clearly distinguished RA serum samples after anti-TNFα/DMARDs combination therapy with high confidence level (*P* <0.05): (hsa-miR-125b, hsa-miR-126-3p, hsa-miR146a-5p, hsa-miR-16-5p, hsa-miR-23-3p, and hsa-miR-223-3p) all of them being increased after treatment (Figure 2A; Tables S3 and S4 in Additional file 1).

These differences were found even more relevant when patients were divided in responders and non-responders, so that while miRNA expression levels strongly increased in responders, they did not change in non-responder patients after anti-TNFα/DMARDs combination therapy (Figure 2B, and Figure S4 in Additional file 2). Moreover, in responders, a parallel change was observed in the expression levels of autoimmune parameters such as the RF and of some cytokines such as TNFα, IL-6 or IL-17, which were found significantly reduced after treatment (Table 2).

### Regulation network of differentially expressed serum miRNAs in the inflammatory pathways and processes of rheumatoid arthritis

**Figure 3:**
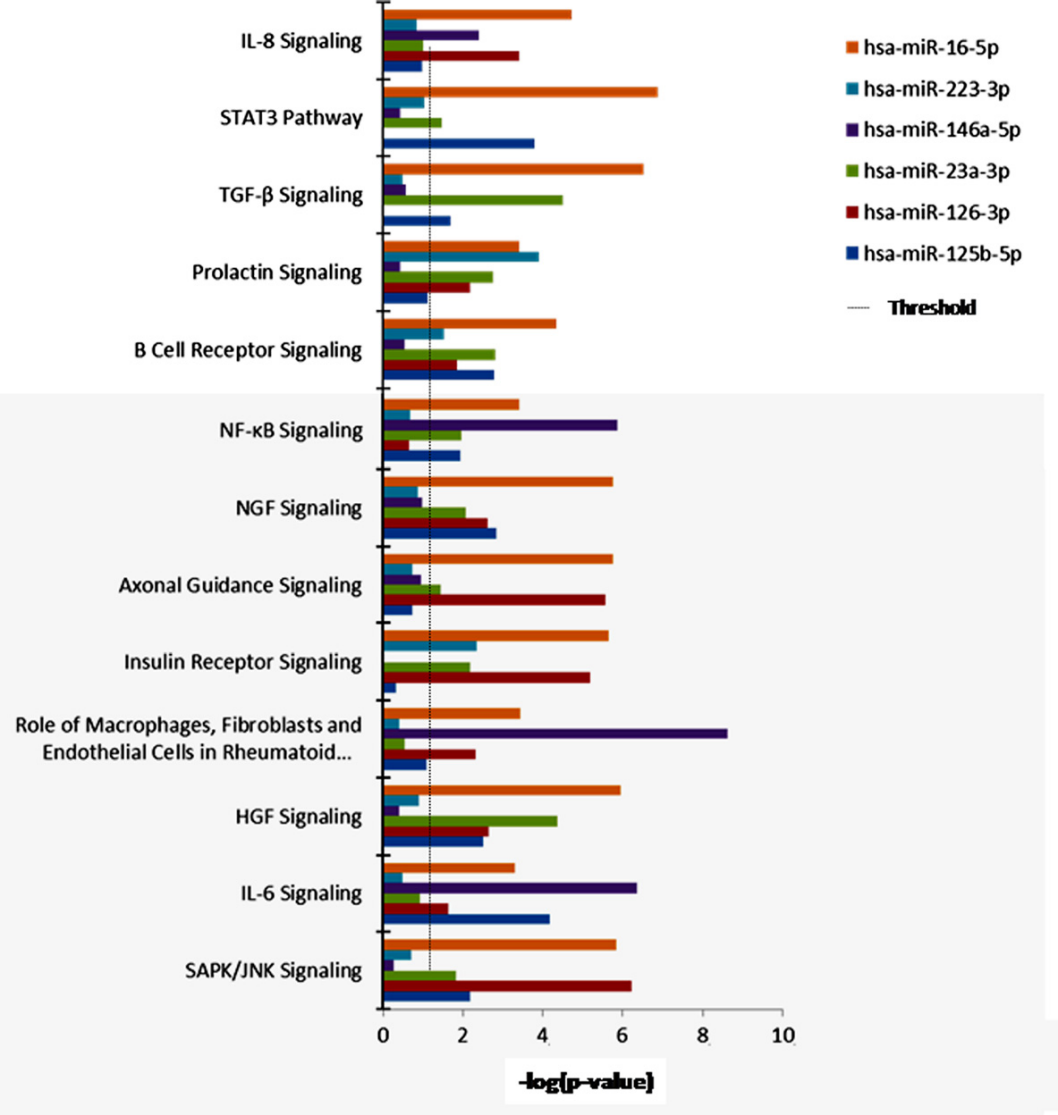
**Participation of the six validated miRNAs in the different canonical pathways.** Ingenuity Pathway Analysis (IPA) uncovered the main enriched biological pathways on which that miRNAs are involved. The analysis included only the pathways with average IPA score >2 (indicated as -log (*P* value)). miRNAs, microRNAs.

It is widely accepted that miRNAs can influence gene expression by causing translational repression or mRNA degradation. This dysregulation can alter several downstream pathways and manifest effects. By using the IPA software, we further investigated the potential gene targets for the six validated miRNAs and analyzed their participation in the different canonical pathways (Figure 3). The study, in which only the pathways with average IPA score >2 (−log (*P* value)) were included, revealed that the most probable genes modified by these miRNA correspond to pathways directly related to RA (that is, the role of macrophages, fibroblasts and endothelial cells in RA, the role of osteoblasts, osteoclasts and chondrocytes in RA, and the role of IL-17A in RA). Pathways related to STAT-3 or IL-6 signaling (both of them crucial for the induction and maintenance of the inflammatory status present in RA patients), were also identified.

To better understand the significance of the results, we investigated the potential impact of the verified miRNAs directly on the RA-related pathways. A deeper analysis of the miRNA targets demonstrated that RA-related canonical pathways may be regulated at different levels (grey-filled symbols of genes in Figure S1 in Additional file 3: Figure S2 in Additional file 4; and Figure S3 in Additional file 5). It is also interesting to note that in this study we found that several genes were the targets of more than one of the verified miRNAs (Table 3). For example, the gene that codifies for conserved helix-loop-helix ubiquitous kinase (CHUK) may be potentially regulated by four out of the six miRNAs. Other genes directly related to RA are multiple targets for these miRNAs, such as IL6 receptor alpha (IL6R) and beta (IL6ST) chains, fibroblast growth factor 2 (FGF2), and the bone morphogenetic protein receptor type II (BMPRII). Interestingly, a number of both miRNA and mRNA targets uncovered in that analysis were found complementary altered after anti-TNFα/DMARDs combination therapy in our patient’s cohort (that is IL-6 or IL-17 serum levels).

**Table 3:** **Potential genes directly related to rheumatoid arthritis that constitute direct targets of the validated miRNAs**

**miR-146**	**miR-223**	**miR-125b**	**miR-126**	**miR-23**	**miR-16**	**Entrez Gene NaME**
***Role of macrophages*** **,** ***fibroblasts and endothelial cells in rheumatoid arthritis***						
**--**	APC	APC	--	--	--	Adenomatous polyposis coli
**--**	--	--	--	CCND1	CCND1	Cyclin D1
**CHUK**	CHUK	--	--	CHUK	CHUK	Conserved helix-loop-helix ubiquitous kinase
**--**	FGF2	--	--	FGF2	FGF2	Fibroblast growth factor 2 (basic)
**--**	FZD4	--	--	FZD4	FZD4	Frizzled class receptor 4
**IL36B**	--	--	--	--	IL36B	Interleukin 36, beta
**IL-36RN**	--	--	--	--	IL36RN	Interleukin 36 receptor antagonist
**--**	--	IL6R	--	IL6R	--	Interleukin 6 receptor
**--**	IL6ST	--	--	IL6ST	--	Interleukin 6 signal transducer
**IRAK2**	--	--	--	--	IRAK2	Interleukin-1 receptor-associated kinase 2
**--**	--	--	LRP6	--	LRP6	Low-density lipoprotein receptor-related protein 6
**--**	PIK3C2A	--	--	PI3KC2A	--	Phosphatidylinositol-4-phosphate 3-kinase, catalytic subunit type 2 alpha
**--**	--	PI3KCD	PI3KCD	--	--	Phosphatidylinositol-4,5-bisphosphate 3-kinase, catalytic subunit delta
**PRKCE**	PRKCE	--	--	PRKCE	--	Protein kinase C, epsilon
***Role of osteoblasts*** **,** ***osteoclasts and chondrocytes in rheumatoid arthritis***						
**miR-146**	**miR-223**	**miR-125b**	**miR-126**	**miR-23**	**miR-16**	**Entrez Gene NaME**
**--**	--	--	--	ADAMTS5	ADAMTS5	ADAM metallopeptidase with thrombospondin type 1 motif, 5
**--**	APC	APC	--	--	--	Adenomatosus polyposis coli
**--**	--	BCL2	--	BCL2	BCL2	B-cell LL/lymphoma 2
**--**	--	BMPR2	--	BMPR2	--	Bone morphogenetic protein receptor, type II (serine/threonine kinase)
**CHUK**	CHUK	--	--	CHUK	CHUK	Conserved helix-loop-helix ubiquitous kinase
**--**	FOXO1	--	--	--	FOXO1	Forkhead box 01
**--**	FZD4	--	--	FZD4	FZD4	Frizzled class receptor 4
**IL1F10**	--	IL1F10	--	--	--	Interleukin 1 family, member 10 (theta)
**IL36B**	--	--	--	--	IL36B	Interleukin 36, beta
**IL36RN**	--	--	--	--	IL36RN	Interleukin 36 receptor antagonist
**--**	--	--	LRP6	--	LRP6	Low-density lipoprotein receptor-related protein 6
**--**	PIK3C2A	--	--	PI3KC2A	--	Phosphatidylinositol-4-phosphate 3-kinase, catalytic subunit type 2 alpha
**--**	--	PI3KCD	PI3KCD	--	--	Phosphatidylinositol-4,5-bisphosphate 3-kinase, catalytic subunit delta
**XIAP**	XIAP	--	--	--	--	X-linked inhibitor of apoptosis
***Role of IL-17A in arthritis***						
**miR-146**	**miR-223**	**miR-125b**	**miR-126**	**miR-23**	**miR-16**	**Entrez Gene NaME**
**PIK3CD**	--	--	PIK3CD	--	--	Phosphatidylinositol-4,5-bisphosphate 3-kinase, catalytic subunit delta

Only those genes regulated by at least two of the validated miRNAs are included in the table. miRNA, microRNA; IL-17A, interleukin 17A.

### Changes in serum miRNAs correlate with changes in clinical variables in RA patients

**Figure 4:**
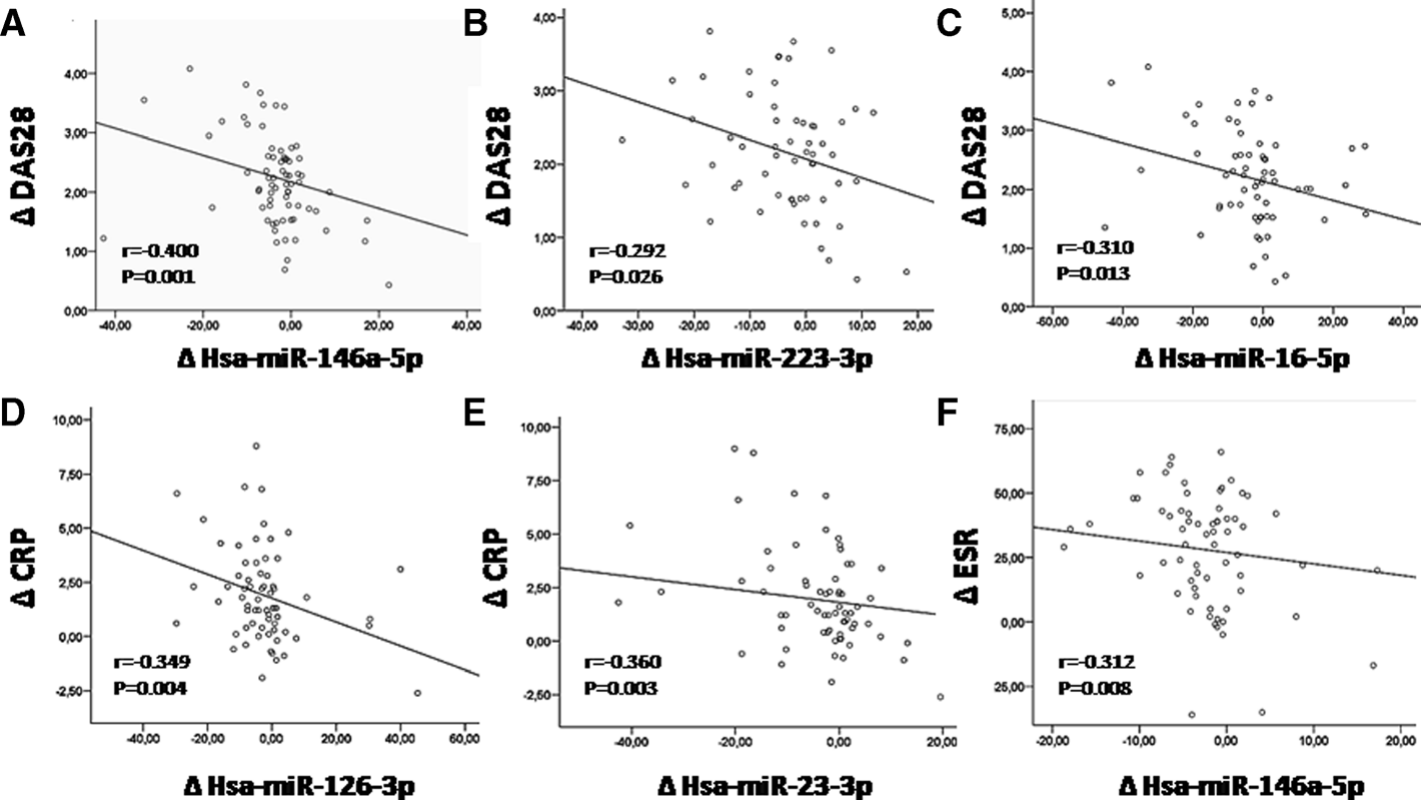
**Changes in serum miRNAs correlate with changes in clinical variables in RA patients.** Changes after anti-TNFα/DMARDs combination therapy (T1-T2) of various miRNAs significantly correlated with the changes in DAS28 **(A-C)**, in CRP **(D-E)** and in ESR **(F)**. r values of Spearman’s rank correlation and *P* values of their null hypothesis are shown. CRP, C-reactive protein; DAS28, disease activity score; DMARDs, disease-modifying antirheumatic drugs; ESR, erythrocyte sedimentation rate; miRNAs, microRNAs; RA, rheumatoid arthritis; T1, baseline; T2, at 6 months; TNFα, tumor necrosis factor alpha.

To assess the possibility of serum miRNAs as biomarkers of RA and of response to therapy, we investigated the correlation of validated miRNAs with clinical and inflammatory variables. The changes observed in three miRNAs (hsa-miR-146a-5p, hsa-miR-223-3p and hsa-miR-16-5p) significantly correlated with the changes observed in clinical parameters (that is, DAS28), and five of them at least with changes in inflammatory parameters such as CRP or ESR (hsa-miR-146a-5p, hsa-miR-223-3p,hsa-miR-16-5p, hsa- miR-126-3p and hsa-miR-23-3p) (Figure 4). A direct and significant relationship was also demonstrated among all the miRNAs (data not shown). In parallel, as described above, IPA analysis showed specific networks demonstrating interrelations among their targets directly associated to RA disease.

### Serum miRNAs hsa-miR-23a-3p and hsa-miR-223-3p as predictors of therapy response in RA patients

**Figure 5:**
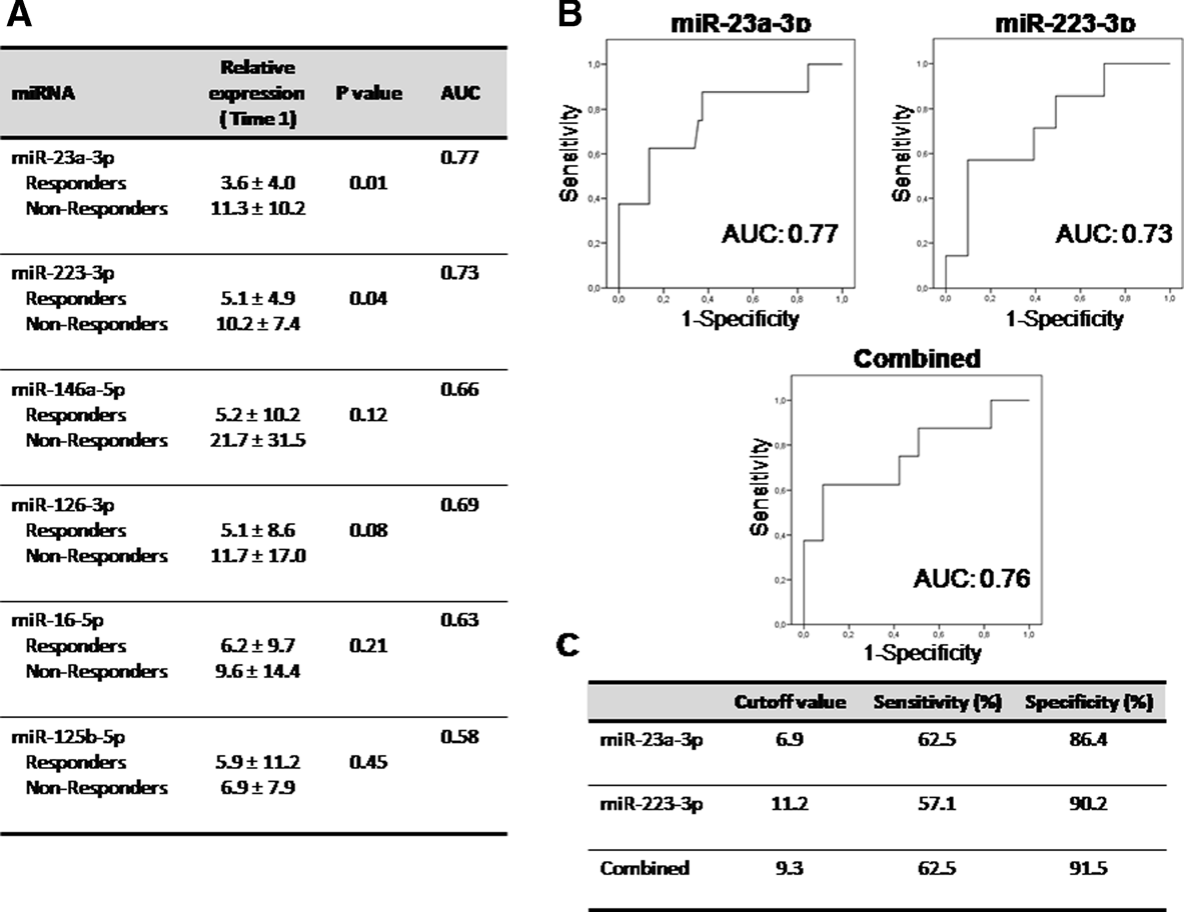
**Evaluation of candidate miRNAs as predictors of response to therapy. (A)** Relative expression levels of the six miRNAs validated in plasma of RA patients (n = 95) before starting the anti-TNFα/DMARDs combination therapy (T1). Data are shown as mean ± standard deviation. Area under the curve (AUC) was calculated after plotting the receiver operating characteristic (ROC) curve. **(B)** ROC curve analyses of miR-23a-3p (left panel) and miR-223-3p (right panel), which showed the highest values for AUC; below is shown the combined panel for the two miRNAs that performed as described in Material and methods. **(C)** Sensitivity and specificity of each miRNA test. Cutoff value with higher specificity was selected. DMARDs, disease-modifying antirheumatic drugs; miRNAs, microRNAs; RA, rheumatoid arthritis; T1, baseline; TNFα, tumor necrosis factor alpha.

As a general feature, we found that the better the status of the patient before the treatment (in terms of clinical and serological parameters) the lowest changes in DAS and levels of miRNAs were found after anti-TNFα/DMARDs combination therapy. In particular, elevated levels of miRNAs before starting the therapy were indicative of no response (Figure 5A).

**Figure 6:**
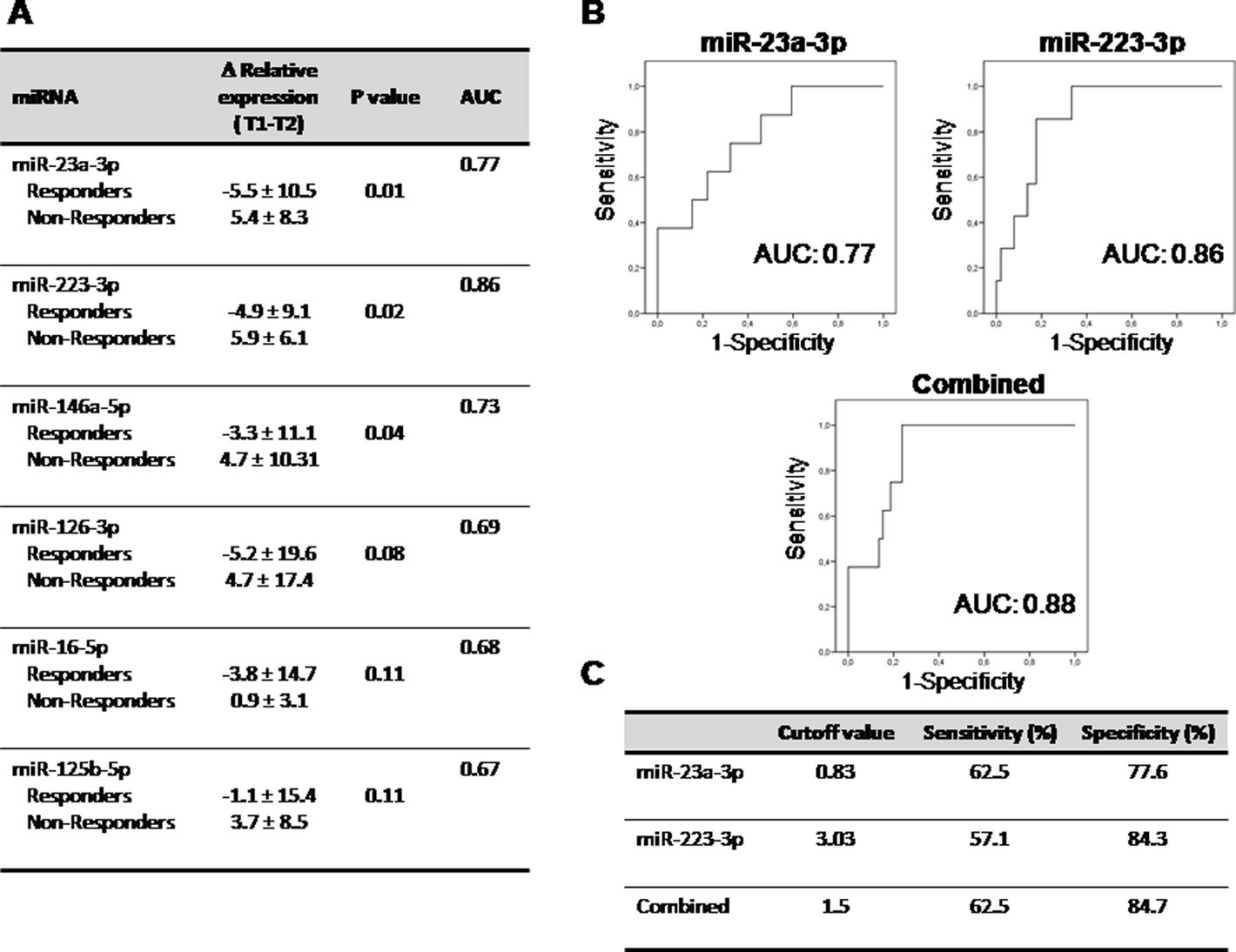
**Evaluation of candidate miRNAs as potential biomarkers of response to anti-TNFα/DMARDs combination therapy. (A)** Changes in relative expression levels of the six miRNAs validated in plasma of RA patients (n = 95) before and after anti-TNFα/DMARDs combination therapy (T1-T2). Data are shown as mean ± standard deviation. Area under the curve (AUC) was calculated after plotting the receiver operating characteristic (ROC) curve. **(B)** ROC curve analyses of miR-23a-3p (left panel) and miR-223-3p (right panel), which showed the highest values for AUC; below is shown the combined panel for the two miRNAs performed as described in Material and methods. **(C)** Sensitivity and specificity of each miRNA test. Cutoff value with higher specificity was selected. DMARDs, disease-modifying antirheumatic drugs; miRNAs, microRNAs; RA, rheumatoid arthritis; T1, baseline; T2, at 6 months; TNFα, tumor necrosis factor alpha.

That data were further supported by ROC analyses, which showed that hsa-miR-23-3p and hsa-miR-223-3p levels at T1, with a cutoff value of 6.9 and 11.2 (relative expression at T1) respectively, were predictors of non-response to anti-TNFα/DMARD combination treatment (Figure 5B-C) with a sensitivity of 62.5% and 57.1%, and a specificity of 86.4% and 90.2% respectively. The analysis of changes in relative expression of miRNAs after treatment further showed a downregulation instead of upregulation in RA patients non-responders, while in responders, a significant increase in three of the miRNAs validated was demonstrated (Figure 6A).

To evaluate their relevance as biomarkers in response to anti-TNFα/DMARDs combination therapy, we conducted a ROC analysis of that miRNAs. ROC analysis showed the highest AUC for miR-23 and miR-223. Changed relative expression between T1 and T2 for miR-23, at a cutoff value of 0.83, demonstrated a sensitivity of 62.5% and a specificity of 77.6%. At a cutoff value of 3.03 for miR-223, the values were 57.1% and 84.3% respectively (Figure 6A-C).

To improve accuracy of the analysis, we performed the combination of ROC curve analyses of miR-23, and miR-223. The ratio of combination for these miRNAs at T1 demonstrated an increase in both the sensitivity (62.5%) and specificity (91.5%) in relation to those given by each miRNA alone (Figure 6B-C). The ratio of combination for the change of these miRNAs (T1-T2) also yielded the highest AUC value of 0.88 and at the optimal cutoff value of 1.5, the sensitivity and specificity were 62.5% and 84.7%, (Figure 6B-C).

Taken together, these results suggest that serum hsa-miR-23a-3p and hsa-miR-223-3p can act both as predictors of therapy response and biomarkers of response to anti-TNFα/DMARDs combination therapy with high specificity.

## Discussion

MicroRNAs are emerging as potential targets for new therapeutic strategies of autoimmune disorders. In the present study, data on miRNA serum levels of RA patients before and after anti-TNFα/DMARDs combination therapy, and their close relationship with the improvement of the disease, suggest their potential use as novel biomarkers for monitoring therapy outcome.

Almost all of the RA patients showed complete clinical response to anti-TNFα/DMARDs combination therapy, not only in disease activity (swollen joints, painful pain scales, DAS28, and so on), but also in physical function, quality of life, fatigue, and sleep (HAQ). These results validate previous studies [20,21].

Most of the miRNAs evaluated - in the setting of our PCR array - were found increased in response to treatment. Since miRNAs generally act as negative regulators of their target proteins, the increase in the levels of miRNAs could imply a reduction in the expression of all their target proteins, which were altered in their expression in RA patients before the biological therapy. In this regard, the functional classification allowed us to demonstrate that the majority of altered miRNAs had as potential target molecules/proteins/transcription factors involved in inflammation and autoimmunity processes, activation of T and B cells, musculoskeletal dysfunction or cardiovascular disease. Therefore, the increase in the levels of those miRNAs after anti-TNFα/DMARDs combination therapy might be associated to a reduction in the inflammatory profile and the improvement of the overall disease status of patients. In support for this hypothesis, we further found a significant reduction in the serum levels of inflammatory and autoimmunity markers.

The proteolytic degradation of extracellular matrix (ECM) molecules in articular cartilage in the joint is a crucial catabolic event in RA [22]. Synoviocytes and synovial macrophages produce inflammatory mediators including prostaglandins, reactive oxygen species and proinflammatory cytokines (such as IL-1ß, IL-6 and TNFα) that stimulate articular chondrocytes to produce matrix-degrading enzymes such as matrix metelloproteinases, leading to the destruction and degeneration of the cartilage ECM [23]. Thus, a putative effect on the reduction of chondrocyte maturation (as identified in the cluster of miRNAs found increased after therapy), might have beneficial effects on the prevention of the articular damage in RA patients.

The miRNAs validated by RT-PCR in our cohort of patients (miR146a-5p, miR-16-5p, miR-23-3p, miR-125b-5p, miR223-3p; miR126-3p) have been previously reported to act as relevant regulators of immune cells development, playing crucial roles in the inflammatory response, and acting as key players in the pathogenesis of various chronic and autoimmune disorders, including RA itself [24].

It is also interesting to note that in this study we found that several genes were the targets of more than one of the verified miRNAs (Table 3). For example, the gene that codifies for CHUK may be potentially regulated by four out of the six miRNAs. CHUK (conserved helix-loop-helix ubiquitous kinase, also known as inhibitor of nuclear factor kappa-B kinase subunit alpha (IKK-α), or IKK1) is a protein kinase that mediates IkappaB phosphorylation and nuclear factor kappa B (NFkB) activation [25]. Almost all of the proinflammatory factors involved in the pathogenesis and progression of RA (that is, IL-6 or TNFα) are regulated by the transcription factor NFkB. Thus, drugs that modulate the activation and function of CHUK are likely to have therapeutic value in inflammatory disease such as RA.

Other genes directly related to RA were also found to be multiple targets for these miRNAs, including IL6 receptor alpha (IL6R) and beta (IL6ST) chains, FGF-2, a number of intracellular molecules, and the BMPR2. Current studies showed that, in addition to their role in enhancing autoantibody production, IL-6 promotes synovial tissue inflammation and osteoclastogenesis, leading to the severe synovitis with pannus formation and the progressive cartilage and bone destruction in multiple joints found in RA [26]. Moreover, IL-6 is an important contributor to the development of cardiovascular disease (CVD) in RA patients [27]. In our cohort, IL-6 receptors alpha and beta were found to be putative targets for three of the six validated miRNAs (hsa-miR-23-3p, hsa-miR-125b-5p, and hsa-miR223-3p). In parallel, plasma analysis showed that anti-TNF drugs promoted a significant reduction on IL-6 levels, thus suggesting a role for those miRNAs in the regulation of IL-6 production.

A direct target for three of the validated miRNAs found altered in RA patients after anti-TNF/DMARDs therapy was the FGF-2. In the synovial fluid FGF2 plays a role in the final step of osteoclastic bone resorption in RA joint destruction that is preceded by recruitment and differentiation of osteoclasts by other factors. Thus, endogenous FGF2 might participate in the pathogenesis of that bone resorptive disease through its direct action on osteoclasts [28].

From a molecular point of view, the severity and prognosis of RA are dependent on the balance between inflammatory or destructive pathways and homeostatic or repair pathways [29]. In that way, class I phosphoinositide 3 kinase (PI3K) δ is a promising therapeutic target for RA. PI3Kδ is highly expressed in RA synovium, especially in the synovial lining. Its expression is selectively induced by the inflammatory cytokines TNF and IL-1. It has been demonstrated that PI3Kδ is a major regulator of platelet-derived growth factor (PDGF)-mediated fibroblast growth and survival via Akt [30]. Thus, targeting PI3Kδ in RA could modulate synoviocyte function via anti-inflammatory and disease-altering mechanisms. Furthermore, the family of PI3Ks plays an important role in the pathogenesis of CVD by modulating several essential biologic processes, such as metabolism, vascular homeostasis and thrombogenicity [31]. In fact, various observations indicate that pharmacological inhibition of PI3Ks may be a new therapeutic strategy for preventing cardiovascular complications in this autoimmune disease.

Increasing evidence suggests a role for bone morphogenetic protein (BMP) signaling in joint homeostasis and disease. BMP signaling, induced through the binding of a dimeric BMP ligand to type I and type II BMP receptors, has a key role in the pathogenesis of RA [32]. Moreover, it has been shown that BMP expression can be regulated by anti-TNFα drugs [33], thus supporting a relevant role for the miRNAs involved in the response to treatment and having that receptor as a target.

Consistent with our results, a recent study has shown the association of two of the miRNAs found significantly increased in response to anti-TNFα/DMARDs combination therapy in our study (hsa-miR-223-3p, and hsa-miR-16-5p) with disease activity in RA patients newly diagnosed [34]. Furthermore, three trials in 2008 indicated the existence of altered expression of some of those miRNAs (hsa-miR-16-3p, hsa-miR-132, hsa-miR-146a-5p and hsa-miR-155-3p) in leukocytes of arthritic patients [35]. A more recent study showed that decreased expression of hsa-miR-146a and hsa-miR-155-3p contributes to an abnormal Treg phenotype in patients with RA [36]. In support for that previously reported data, correlation studies in our cohort demonstrated, first, the existence of a significant relationship among all the validated miRNAs. Moreover, all of them have putative targets directly associated to RA disease and involved in the response to anti-TNFα drugs.

Second, we found a negative correlation between the changes in the expression levels of almost all the validated miRNAs and the changes occurred in various clinical and inflammatory parameters. Furthermore, ROC analyses demonstrated that two of these six miRNAs (hsa-miR-23-3p and hsa-miR-223-3p) can act in RA patients as both predictors of therapy response (indicating those patients who would not benefit from anti-TNFα/DMARDs combination therapy), and as biomarkers of response to anti-TNFα/DMARDs combination therapy (so that their levels would be indicative of treatment efficacy and also of the degree of response).

Our data contrast with a recent study performed in patients with psoriasis treated with the TNFα-inhibitor etanercept [37]. In that cohort of patients, etanercept significantly downregulated serum levels of hsa-miR-223-3p and hsa-miR-126-5p among others. In addition, those miRNAs were not related to disease severity in psoriasis. Those results suggest a distinctive involvement of similar miRNAs in pathways affected by anti-TNFα/DMARDs combination therapy depending on the inflammatory disease concerned.

## Conclusions

Altogether, our data suggest that differentially expressed miRNAs in the serum of RA patients before and after anti-TNFα/DMARDs combination therapy have potential to serve as novel biomarkers for predicting and monitoring therapy outcome.

Since we did not perform a complete plasma human microarray analysis, we cannot exclude the complementary role of other circulating miRNAs in the response to treatment, and because of the clinical heterogeneity of RA patients, our data must be confirmed in larger studies. Moreover, specific studies on the mechanisms underlying the altered expression of those miRNAs after anti-TNFα/DMARDs combination therapy, as well as the identification of the mechanism and cellular sources of those extracellular miRNAs are still required.

## Additional files

Additional file 1: Table S1. Changes operated on clinical and serological parameters after anti-TNFα/DMARDs combination therapy. **Table S2.** Comparative analysis among groups of treatment. Validation cohort. **Table S3.** Changes operated on validated miRNAs after anti-TNFα/DMARDs combination therapy. **Table S4.** Comparative analysis among groups of treatment. **Table S5.** Individual patient’s treatment. Additional file 2: Figure S4. Relative miRNA levels at starting (T1) and after six months of anti-TNFα/DMARDs combination therapy (T2) in the validation cohort (n = 85). To validate the PCR array data,10 miRNAs differentially expressed were selected (hsa-miR-125b, hsa-miR-23a-3p, hsa-miR-21-5p, hsa-miR-126-3p, hsa-miR-146a-5p, hsa-let-7a-5p, hsa-miR-16-5p, hsa-miR-124a-3p, hsa-miR-155-5p, and hsa-miR-223). (A) Relative expression levels of each miRNA in the group of RA patients responders to therapy (n = 75). Boxes indicate the interval between the 25^th^ and 75^th^ percentiles and horizontal bars inside boxes indicate median. Whiskers indicate the interval of data within 1.5 × interquartile ranges (IQR). Closed circles indicate data points outside 1.5 × IQR. ^*^ *P* < 0.05. (B) Relative expression levels of each miRNA in the group of non-responders to the combination therapy (n = 10). Additional file 3: Figure S1. Distribution of all the genes potentially modified by the validated miRNAs integrated in the pathways related to the role of macrophages, fibroblasts and endothelial cells in rheumatoid arthritis. The different points were RA-related canonical pathways might be regulated are represented by grey-filled symbols. Additional file 4: Figure S2. Distribution of all the genes potentially modified by the validated miRNAs integrated in the pathways related to the role of osteoblasts, osteoclasts, and chondrocytes in rheumatoid arthritis. The different points were RA-related canonical pathways might be regulated are represented by grey-filled symbols. Additional file 5: Figure S3. Distribution of all the genes potentially modified by the validated miRNAs integrated in the pathways related to the role of IL-17A in rheumatoid arthritis. The different points were RA-related canonical pathways might be regulated are represented by grey-filled symbols.

## Abbreviations

ADA: adalimumab
ADAMTS5: ADAM metallopeptidase with thrombospondin type 1 motif 5
anti-CCPs: anti-cyclic citrullinated peptides
APC: adenomatous polyposis coli
AUCs: areas under the curve
BCL1: B-cell CLL/Lymphoma 2
BMP: bone morphogenetic protein
BMPRII: bone morphogenetic protein receptor type II
CCND1: cyclin D1
CHUK: conserved helix-loop-helix ubiquitous kinase
CRP: C-reactive protein
CVD: cardiovascular disease
DAS28: disease activity score
DMARDs: disease-modifying antirheumatic drugs
ECM: extracellular matrix
ELISA: enzyme-linked immunosorbent assay
ESR: erythrocyte sedimentation rate
ETA: etanercept
FGF2: fibroblast growth factor 2
FOXO1: forkhead box 01
FZD4: frizzled class receptor 4
HAQ: health assessment questionnaire
HCQ: hydroxychloroquine
IFX: infliximab
IKKα: inhibitor of nuclear factor kappa-B kinase subunit alpha
IL: interleukin
IL6R: IL-6 receptor
IL6ST: IL-6 signal transducer
IPA: Ingenuity Pathway Analysis
IRAK2: interleukin-1 receptor-associated kinase 2
LRP6: low-density lipoprotein receptor-related protein 6
MCP-1: monocyte chemotactic protein
miRNAs: microRNAs
mRNA: messenger RNA
miRTC: miRNA reverse transcription control assay
NFkB: nuclear factor kappa B
NSAIDs: nonsteroidal anti-inflammatory drugs
PDGF: platelet-derived growth factor
PIK3C2A: phosphatidylinositol-4-phosphate 3-kinase, catalytic subunit type 2 alpha
PIK3CD: phosphatidylinositol-4,5 biphosphate 3-kinase, catalytic subunit delta
PPC: positive PCR control
PRKCE: protein kinase C, epsilon
RA: rheumatoid arthritis
RF: rheumatoid factor
ROC curve: receiver operating characteristic curve
SD: standard deviation
SDAI: simple disease activity index
SJC: swollen joint count
sTNFRII: soluble TNF receptor II
T1: baseline
T2: at 6 months
Th-17: T helper 17
TJC: tender joint count
TNFα: tumor necrosis factor type alpha
VAS: visual analog scale of pain
VEGF: vascular endothelial growth factor
XIAP: X-linked inhibitor of apoptosis

## References

[1] Filková M, Jüngel A, Gay RE, Gay S (2012). MicroRNAs in rheumatoid arthritis: potential role in diagnosis and therapy. BioDrugs..

[2] Atzeni F, Sarzi-Puttini P (2009). Anti-cytokine antibodies for rheumatic diseases. Curr Opin Investig Drugs..

[3] Choy EH, Panayi GS (2001). Cytokine pathways and joint inflammation in rheumatoid arthritis. N Engl J Med..

[4] Rubbert-Roth A, Finckh A (2009). Treatment options in patients with rheumatoid arthritis failing initial TNF inhibitor therapy: a critical review. Arthritis Res Ther..

[5] Prajapati R, Plant D, Barton A (2011). Genetic and genomic predictors of anti-TNF response. Pharmacogenomics..

[6] Plenge RM, Criswell LA (2008). Genetic variants that predict response to anti-tumor necrosis factor therapy in rheumatoid arthritis: current challenges and future directions. Curr Opin Rheumatol..

[7] Krintel SB, Grunert VP, Hetland ML, Johansen JS, Rothfuss M, Palermo G (2013). The frequency of anti-infliximab antibodies in patients with rheumatoid arthritis treated in routine care and the associations with adverse drug reactions and treatment failure. Rheumatology (Oxford)..

[8] Ortea I, Roschitzki B, Ovalles JG, Longo JL, de la Torre I, González I (2012). Discovery of serum proteomic biomarkers for prediction of response to infliximab (a monoclonal anti-TNF antibody) treatment in rheumatoid arthritis: an exploratory analysis. J Proteomics..

[9] Bottini N, Firestein GS (2013). Epigenetics in rheumatoid arthritis: a primer for rheumatologists. Curr Rheumatol Rep..

[10] Ceribelli A, Yao B, Dominguez-Gutierrez PR, Nahid MA, Satoh M, Chan EK (2011). MicroRNAs in systemic rheumatic diseases. Arthritis Res Ther..

[11] Pauley KM, Satoh M, Chan AL, Bubb MR, Reeves WH, Chan EK (2008). Upregulated miR-146a expression in peripheral blood mononuclear cells from rheumatoid arthritis patients. Arthritis Res Ther..

[12] Niimoto T, Nakasa T, Ishikawa M, Okuhara A, Izumi B, Deie M (2010). MicroRNA-146a expresses in interleukin-17 producing T cells in rheumatoid arthritis patients. BMC Musculoskelet Disord..

[13] Stanczyk J, Pedrioli DM, Brentano F, Sanchez-Pernaute O, Kolling C, Gay RE (2008). Altered expression of MicroRNA in synovial fibroblasts and synovial tissue in rheumatoid arthritis. Arthritis Rheum..

[14] Nakamachi Y, Kawano S, Takenokuchi M, Nishimura K, Sakai Y, Chin T (2009). MicroRNA-124a is a key regulator of proliferation and monocyte chemoattractant protein 1 secretion in fibroblast-like synoviocytes from patients with rheumatoid arthritis. Arthritis Rheum..

[15] Nakasa T, Shibuya H, Nagata Y, Niimoto T, Ochi M (2011). The inhibitory effect of microRNA-146a expression on bone destruction in collagen-induced arthritis. Arthritis Rheum..

[16] Murata K, Yoshitomi H, Tanida S, Ishikawa M, Nishitani K, Ito H (2010). Plasma and synovial fluid microRNAs as potential biomarkers of rheumatoid arthritis and osteoarthritis. Arthritis Res Ther..

[17] Aletaha D, Neogi T, Silman AJ, Funovits J, Felson DT, Bingham CO (2010). Rheumatoid arthritis classification criteria: an American College of Rheumatology/European League Against Rheumatism collaborative initiative. Arthritis Rheum..

[18] Chen JS, Makovey J, Lassere M, Buchbinder R, March LM (2014). Comparative effectiveness of anti-tumour necrosis factor (TNF) drugs on health-related quality of life among patients with inflammatory arthritis. Arthritis Care Res (Hoboken)..

[19] Pfaffl MW, Tichopad A, Prgomet C, Neuvians TP (2004). Determination of stable housekeeping genes, differentially regulated target genes and sample integrity: BestKeeper – Excel-based tool using pair-wise correlations. Biotechnol Lett..

[20] Flouri I, Markatseli TE, Voulgari PV, Boki KA, Papadopoulos I, Settas L (2014). Comparative effectiveness and survival of infliximab, adalimumab, and etanercept for rheumatoid arthritis patients in the Hellenic Registry of Biologics: low rates of remission and 5-year drug survival. Semin Arthritis Rheum..

[21] Goldring MB, Marcu KB (2009). Cartilage homeostasis in health and rheumatic diseases. Arthritis Res Ther..

[22] Csaki C, Mobasheri A, Shakibaei M (2009). Synergistic chondroprotective effects of curcumin and resveratrol in human articular chondrocytes: inhibition of IL-1beta-induced NF-kappaB-mediated inflammation and apoptosis. Arthritis Res Ther..

[23] Wang H, Peng W, Ouyang X, Li W, Dai Y (2012). Circulating microRNAs as candidate biomarkers in patients with systemic lupus erythematosus. Transl Res..

[24] Luo X, Ranade K, Talker R, Jallal B, Shen N, Yao Y (2013). microRNA-mediated regulation of innate immune response in rheumatic diseases. Arthritis Res Ther..

[25] Mercurio F, Zhu H, Murray BW, Shevchenko A, Bennett BL, Li J (1997). IKK-1 and IKK-2: cytokine-activated IkappaB kinases essential for NF-kappaB activation. Science..

[26] Ohtsuji M, Lin Q, Nishikawa K, Ohtsuji N, Okazaki H, Tsurui H (2015). IL-6 signal blockade ameliorates the enhanced osteoclastogenesis and the associated joint destruction in a novel FcγRIIB-deficient rheumatoid arthritis mouse model. Mod Rheumatol..

[27] Panoulas VF, Stavropoulos-Kalinoglou A, Metsios GS, Smith JP, Milionis HJ, Douglas KM (2009). Association of interleukin-6 (IL-6)-174G/C gene polymorphism with cardiovascular disease in patients with rheumatoid arthritis: the role of obesity and smoking. Atherosclerosis..

[28] Chikazu D, Hakeda Y, Ogata N, Nemoto K, Itabashi A, Takato T (2000). Fibroblast growth factor (FGF)-2 directly stimulates mature osteoclast function through activation of FGF receptor 1 and p42/p44 MAP kinase. J Biol Chem..

[29] Luyten FP, Lories RJ, Verschueren P, de Vlam K, Westhovens R (2006). Contemporary concepts of inflammation, damage and repair in rheumatic diseases. Best Pract Res Clin Rheumatol..

[30] Bartok B, Boyle DL, Liu Y, Ren P, Ball ST, Bugbee WD (2012). PI3 kinase δ is a key regulator of synoviocyte function in rheumatoid arthritis. Am J Pathol..

[31] Eisenreich A, Rauch U (2011). PI3K inhibitors in cardiovascular disease. Cardiovasc Ther..

[32] Lories RJ, Daans M, Derese I, Matthys P, Kasran A, Tylzanowski P (2006). Noggin haploinsufficiency differentially affects tissue responses in destructive and remodeling arthritis. Arthritis Rheum..

[33] Korkosz M, Gąsowski J, Leszczyński P, Pawlak-Buś K, Jeka S, Siedlar M (2014). Effect of tumour necrosis factor-α inhibitor on serum level of dickkopf-1 protein and bone morphogenetic protein-7 in ankylosing spondylitis patients with high disease activity. Scand J Rheumatol..

[34] Filková M, Aradi B, Senolt L, Ospelt C, Vettori S, Mann H (2014). Association of circulating miR-223 and miR-16 with disease activity in patients with early rheumatoid arthritis. Ann Rheum Dis..

[35] Pauley KM, Chan EK (2008). MicroRNAs and their emerging roles in immunology. Ann N Y Acad Sci..

[36] Zhou Q, Haupt S, Kreuzer JT, Hammitzsch A, Proft F, Neumann C, et al. Decreased expression of miR-146a and miR-155 contributes to an abnormal Treg phenotype in patients with rheumatoid arthritis. Ann Rheum Dis. 2014; doi:10.1136/annrheumdis-2013-204377. [Epub ahead of print]. 10.1136/annrheumdis-2013-204377 24562503

[37] Pivarcsi A, Meisgen F, Xu N, Ståhle M, Sonkoly E (2013). Changes in the level of serum microRNAs in patients with psoriasis after antitumour necrosis factor-α therapy. Br J Dermatol..

