# Supplementary material for: Circulating miRNAs as potential biomarkers of therapy effectiveness in rheumatoid arthritis patients treated with anti-TNFα
Source: Arthritis Res Ther. 2015 Mar 9;17(1):49. doi: 10.1186/s13075-015-0555-z (PMC4377058; doi:10.1186/s13075-015-0555-z)
Supplement: Additional file 1: Table S1. — Changes operated on clinical and serological parameters after anti-TNFα/DMARDs combination therapy. Table S2. Comparative analysis among groups of treatment. Validation cohort. Table S3. Changes operated on validated miRNAs after anti-TNFα/DMARDs combination therapy. Table S4. Comparative analysis among groups of treatment. Table S5. Individual patient’s treatment. [file 13075_2015_555_MOESM1_ESM.doc]

**Additional file 1**

**Table S1. Changes operated on clinical and serological parameters after anti-TNFα/DMARDs combination therapy.**

**Validation cohort**

|  | | **TJC** | **SJC** | **DAS28** | **SDAI** | **HAQ** | **ESR**  **(mm/h)** | **CRP**  **(mg/L)** | **RF**  **(U/L)** |
| --- | --- | --- | --- | --- | --- | --- | --- | --- | --- |
| All the patients | T1 | 15.7±4.6 | 11.5±3.8 | 5.7±0.6 | 36.3±10.9 | 2.1±0.3 | 55.9±16.6 | 3.8±2.1 | 114.1±172.5 |
|  | T2 | 5.5±2.6 | 3.2±2.2. | 3.4±0.8 | 3.4±7.6 | 1±0.4 | 29.4±19.4 | 1.6±1.2 | 52.9±108.4 |
|  | *P** | *<0.001* | *<0.001* | *<0.001* | *<0.001* | *<0.001* | *<0.001* | *<0.001* | *<0.001* |
| Patients treated with **INFLIXIMAB**  (N=46) | T1 | 15.4±4.3 | 11.1 ±3.6 | 5.7±0.5 | 35.3±10.1 | 2.1±0.2 | 56±17.7 | 3.9±2.2 | 100±148.1 |
|  | T2 | 5.5±2.4 | 3±1.9 | 3.5±0.7 | 2.6±6.6 | 1±0.3 | 28.5±18.9 | 1.6±1.3 | 41.9±76.8 |
|  | *P*** | *<0.001* | *<0.001* | *<0.001* | *<0.001* | *<0.001* | *<0.001* | *<0.001* | *<0.001* |
| Patients treated with **ETANERCEPT**  (N=24) | T1 | 16.9±5.1 | 12.6±4.1 | 5.8±0.6 | 38.3±12.4 | 2±0.4 | 55.6±14.3 | 3.9±2.2 | 169.3±241.5 |
|  | T2 | 5.7±2.7 | 3.7±2.5 | 3.6±0.9 | 5±9.8 | 1.1±0.4 | 25.4±16.8 | 1.6±1.3 | 85.9±165.1 |
|  | *P*** | *<0.001* | *<0.001* | *<0.001* | *<0.001* | *<0.001* | *<0.001* | *<0.001* | *<0.001* |
| Patients treated with **ADALIMUMAB**  (N=15) | T1 | 14.8±4.2 | 10.9±3.6 | 5.5±0.6 | 35.8±11.1 | 2±0.4 | 56.2±18.3 | 3.2±1.9 | 68.8±64.2 |
|  | T2 | 5.1±3 | 2.8±2.4 | 3.2±0.9 | 3.5±7 | 0.9±4.8 | 38.4±23 | 1.4±1.1 | 33.6±63.9 |
|  | *P*** | *<0.001* | *<0.001* | *<0.001* | *<0.001* | *<0.001* | *0.003* | *0.006* | *0.160* |

*p<0.05 vs T1 (Wilcoxon test); **p<0.05 vs T1 (repeated measures ANOVA)

Patients at baseline (T1) and 6 months after treatment (T2)

**Table S2. Comparative analysis among groups of treatment. Validation cohort**

|  | **TJC** | **SJC** | **DAS28** | **SDAI** | **HAQ** | **ESR**  **(mm/h)** | **CRP**  **(mg/L)** | **RF**  **(U/L)** |
| --- | --- | --- | --- | --- | --- | --- | --- | --- |
| **Infliximab vs Etanercept*** | 0.920 | 0.406 | 0.810 | 0.283 | 0.726 | 0.531 | 0.955 | 0.645 |
| **Infliximab vs Adalimumab*** | 0.699 | 0.739 | 0.557 | 0.707 | 0.717 | 0.081 | 0.500 | 0.608 |
| **Etanercept vs Adalimumab*** | 0.788 | 0.351 | 0.480 | 0.625 | 0.547 | 0.051 | 0.570 | 0.916 |

*p<0.05 (repeated measures ANOVA)

**Table S3. Changes operated on validated miRNAs after anti-TNFα/DMARDs combination therapy.**

**Validation cohort**

| **miRNA** | | **hsa-miR-125b** | **hsa-miR-**  **23a-3p** | **hsa-miR-**  **126-3p** | **hsa-miR-**  **146a-5p** | **hsa-miR-**  **16-5p** | **hsa-miR-**  **223-3p** |
| --- | --- | --- | --- | --- | --- | --- | --- |
| **All the patients** | T1 | 4.5±5.2 | 4.8±5.9 | 5.9±10.1 | 5.4±11.8 | 5.7±9.2 | 5.6±5.6 |
|  | T2 | 11.4±32.3 | 11.6±18.3 | 11.2±18.4 | 8.5±9.1 | 9.5±12.1 | 9.7±8.6 |
|  | *P** | *0.009* | *0.007* | *0.001* | *0.001* | *0.017* | *0.014* |
| **Patients treated with INFLIXIMAB**  **(N=46)** | T1 | 5.6±6.1 | 5.4 ±5.1 | 4.1±4.4 | 5.7±15.7 | 6.9±9.4 | 7.3±6.4 |
|  | T2 | 10.3±11.5 | 14.8±21.7 | 14.1±23.1 | 9.3±8.8 | 12.3±11.1 | 12.1±8.2 |
|  | *P^**^* | *0.421* | *0.014* | *0.008* | *0.146* | *0.058* | *0.019* |
| **Patients treated with ETANERCEPT**  **(N=24)** | T1 | 1.9±2.7 | 1.6±1.5 | 4.2±10.3 | 3.6±4.6 | 2.2±5.4 | 2.8±2.7 |
|  | T2 | 2.9±3.2 | 4.8±10.7 | 4.7±4.8 | 5.9±7.7 | 3.1±4.9 | 5.8±7.3 |
|  | *P^**^* | *0.888* | *0.607* | *0.919* | *0.479* | *0.827* | *0.236* |
| **Patients treated with ADALIMUMAB**  **(N=15)** | T1 | 5.6±4.5 | 8.9±9.2 | 13.7±16.6 | 8.7±6.3 | 8.7±12.8 | 5.6±5.8 |
|  | T2 | 17.4±11.9 | 14.8±17.2 | 13.8±16.6 | 12.3±11.6 | 13.9±18.6 | 10.3±10.5 |
|  | *P^**^* | *0.022* | *0.305* | *0.984* | *0.944* | *0.732* | *0.684* |

*p<0.05 vs T1 (Wilcoxon test); **p<0.05 vs T1 (repeated measures ANOVA)

Patients at baseline (T1) and 6 months after treatment (T2)

**Table S4. Comparative analysis among groups of treatment. Validation cohort**

| **miRNA** | **hsa-miR-125b** | **hsa-miR-**  **23a-3p** | **hsa-miR-**  **126-3p** | **hsa-miR-**  **146a-5p** | **hsa-miR-**  **16-5p** | **hsa-miR-**  **223-3p** |
| --- | --- | --- | --- | --- | --- | --- |
| **Infliximab vs Etanercept*** | 0.434 | 0.067 | 0.091 | 0.194 | 0.009 | 0.021 |
| **Infliximab vs Adalimumab*** | 0.117 | 0.945 | 0.842 | 0.249 | 0.793 | 0.378 |
| **Etanercept vs Adalimumab*** | 0.045 | 0.184 | 0.285 | 0.047 | 0.027 | 0.325 |

*P<0.05 (repeated measures ANOVA)

**Table S5. Individual patient’s treatment**

| **Patient** | **Anti-TNF drug** | **Age**  **(years)** | **Number of previous DMARs** | **DMARDs** | **Number of DMARDs at baseline** | **MTX**  **dose**  **mg/week** | **LFN**  **dose**  **mg/day** | **CyA**  **dose**  **mg/day** | **AZA**  **mg/day** | **HCQ**  **mg/day** | **SZZ**  **mg/day** | **Steroid dose*** | **NSAIDs daily** |
| --- | --- | --- | --- | --- | --- | --- | --- | --- | --- | --- | --- | --- | --- |
| 1 | Infliximab | 62 | 2 | MTX | 1 | 20 |  |  |  |  |  | 7.5 | yes |
| 2 | Etanercept | 54 | 6 | MTX | 1 | 20 |  |  |  |  |  | 0.0 | yes |
| 3 | Infliximab | 42 | 2 | MTX | 1 | 20 |  |  |  |  |  | 0.0 | yes |
| 4 | Infliximab | 59 | 4 | LFN | 1 |  | 20 |  |  |  |  | 10.0 | yes |
| 5 | Infliximab | 55 | 3 | CyA | 1 |  |  | 200 |  |  |  | 5.0 | yes |
| 6 | Adalimumab | 57 | 2 | LFFN | 1 |  | 20 |  |  |  |  | 7.5 | yes |
| 7 | Infliximab | 44 | 4 | LFN + HCQ | 2 |  | 20 |  |  | 400 |  | 5.0 | yes |
| 8 | Infliximab | 57 | 3 | LFN | 1 |  | 20 |  |  |  |  | 10.0 | yes |
| 9 | Infliximab | 56 | 3 | MTX+LFN | 2 | 20 | 20 |  |  |  |  | 5.0 | yes |
| 10 | Infliximab | 55 | 2 | MTX+HCQ | 2 | 20 |  |  |  | 400 |  | 0.0 | yes |
| 11 | Etanercept | 61 | 2 | MTX+HCQ | 2 | 15 |  |  |  | 400 |  | 0.0 | yes |
| 12 | Etanercept | 61 | 3 | MTX | 1 | 20 |  |  |  |  |  | 0.0 | yes |
| 13 | Infliximab | 29 | 2 | MTX+HCQ | 2 | 15 |  |  |  | 400 |  | 0.0 | yes |
| 14 | Infliximab | 57 | 2 | MTX+HCQ | 2 | 15 |  |  |  | 400 |  | 0.0 | yes |
| 15 | Etanercept | 50 | 2 | MTX | 1 | 20 |  |  |  |  |  | 7.5 | yes |
| 16 | Infliximab | 65 | 2 | MTX+HCQ | 2 | 15 |  |  |  | 400 |  | 10.0 | yes |
| 17 | Infliximab | 65 | 4 | AZA+LFN | 2 |  | 20 |  | 100 |  |  | 0.0 | yes |
| 18 | Adalimumab | 56 | 2 | MTX | 1 | 20 |  |  |  |  |  | 0.0 | yes |
| 19 | Infliximab | 61 | 4 | MTX | 1 | 20 |  |  |  |  |  | 10.0 | yes |
| 20 | Etanercept | 25 | 2 | MTX | 1 | 20 |  |  |  |  |  | 7.5 | yes |
| 21 | Infliximab | 60 | 3 | MTX+HCQ | 2 | 15 |  |  |  | 400 |  | 0.0 | yes |
| 22 | Infliximab | 35 | 2 | MTX | 1 | 20 |  |  |  |  |  | 7.5 | yes |
| 23 | Infliximab | 33 | 3 | MTX | 1 | 20 |  |  |  |  |  | 0.0 | yes |
| 24 | Infliximab | 40 | 2 | MTX | 1 | 20 |  |  |  |  |  | 5.0 | yes |
| 25 | Etanercept | 62 | 2 | AZA | 1 |  |  |  | 150 |  |  | 0.0 | yes |
| 26 | Etanercept | 65 | 3 | MTX+LFN | 2 | 20 | 20 |  |  |  |  | 7.5 | yes |
| 27 | Etanercept | 54 | 3 | MTX+LFN | 2 | 20 | 20 |  |  |  |  | 0.0 | yes |
| 28 | Adalimumab | 53 | 2 | MTX+HCQ | 2 | 15 |  |  |  | 400 |  | 0.0 | yes |
| 29 | Etanercept | 63 | 3 | LFN | 1 |  | 20 |  |  |  |  | 5.0 | yes |
| 30 | Infliximab | 63 | 2 | MTX+LFN | 2 | 20 | 20 |  |  |  |  | 7.5 | yes |
| 31 | Infliximab | 67 | 2 | MTX | 1 | 20 |  |  |  |  |  | 0.0 | yes |
| 32 | Adalimumab | 29 | 2 | LFN | 1 |  | 20 |  |  |  |  | 15.0 | yes |
| 33 | Etanercept | 59 | 3 | MTX | 1 | 20 |  |  |  |  |  | 7.5 | yes |
| 34 | Infliximab | 74 | 4 | MTX | 1 | 20 |  |  |  |  |  | 0.0 | yes |
| 35 | Etanercept | 59 | 2 | MTX | 1 | 20 |  |  |  |  |  | 5.0 | yes |
| 36 | Adalimumab | 37 | 3 | MTX+HCQ | 2 | 15 |  |  |  | 400 |  | 5.0 | yes |
| 37 | Infliximab | 62 | 4 | MTX+LFN | 2 | 20 | 20 |  |  |  |  | 7.5 | yes |
| 38 | Infliximab | 61 | 3 | AZAa+LFN | 2 |  | 20 |  | 100 |  |  | 0.0 | yes |
| 39 | Infliximab | 58 | 3 | MTX+LFN | 2 | 20 | 20 |  |  |  |  | 0.0 | yes |
| 40 | Infliximab | 58 | 2 | MTX+LFN | 2 | 20 | 20 |  |  |  |  | 5.0 | yes |
| 41 | Adalimumab | 58 | 2 | MTX | 1 | 20 |  |  |  |  |  | 5.0 | yes |
| 42 | Adalimumab | 56 | 4 | AZA | 1 |  |  |  | 100 |  |  | 10.0 | yes |
| 43 | Etanercept | 45 | 3 | LFN+HCQ | 2 |  | 20 |  |  | 400 |  | 10.0 | yes |
| 44 | Infliximab | 26 | 2 | MTX+HCQ | 2 | 20 |  |  |  | 400 |  | 0.0 | yes |
| 45 | Infliximab | 58 | 2 | MTX | 1 | 20 |  |  |  |  |  | 0.0 | yes |
| 46 | Infliximab | 56 | 4 | MTX+LFN | 2 | 20 | 20 |  |  |  |  | 10.0 | yes |
| 47 | Etanercept | 28 | 2 | MTX+LFN | 2 | 20 | 20 |  |  |  |  | 10.0 | yes |
| 48 | Etanercept | 24 | 3 | MTX | 1 | 20 |  |  |  |  |  | 0.0 | yes |
| 49 | Adalimumab | 37 | 2 | MTX+HCQ | 2 | 20 |  |  |  | 400 |  | 7.5 | yes |
| 50 | Infliximab | 49 | 3 | MTX+HCQ | 2 | 20 |  |  |  | 400 |  | 5.0 | yes |
| 51 | Etanercept | 61 | 5 | MTX+LFN | 2 | 20 | 20 |  |  |  |  | 5.0 | yes |
| 52 | Infliximab | 56 | 4 | LFN+HCQ | 2 |  | 20 |  |  | 400 |  | 10.0 | yes |
| 53 | Infliximab | 43 | 2 | MTX+HCQ | 2 | 20 |  |  |  | 400 |  | 0.0 | yes |
| 54 | Etanercept | 54 | 2 | MTX | 1 | 20 |  |  |  |  |  | 5.0 | yes |
| 55 | Infliximab | 51 | 3 | MTX+HCQ | 2 | 20 |  |  |  | 400 |  | 7.5 | yes |
| 56 | Adalimumab | 62 | 2 | MTX | 1 | 20 |  |  |  |  |  | 7.5 | yes |
| 57 | Infliximab | 53 | 3 | MTX+HCQ | 2 | 15 |  |  |  | 400 |  | 0.0 | yes |
| 58 | Infliximab | 60 | 1 | LFN+SSZ | 2 |  | 20 |  |  |  | 2000 | 7.5 | yes |
| 59 | Adalimumab | 63 | 6 | LFN+SSZ | 2 |  | 20 |  |  |  | 3000 | 5.0 | yes |
| 60 | Infliximab | 50 | 2 | CyA | 1 |  |  | 100 |  |  |  | 0.0 | yes |
| 61 | Etanercept | 46 | 2 | MTX | 1 | 20 |  |  |  |  |  | 0.0 | yes |
| 62 | Etanercept | 40 | 2 | MTX | 1 | 20 |  |  |  |  |  | 0.0 | yes |
| 63 | Infliximab | 42 | 2 | MTX+LFN | 2 | 20 | 20 |  |  |  |  | 0.0 | yes |
| 64 | Adalimumab | 53 | 1 | LFN | 1 |  | 20 |  |  |  |  | 7.5 | yes |
| 65 | Infliximab | 63 | 2 | MTX | 1 | 20 |  |  |  |  |  | 7.5 | yes |
| 66 | Infliximab | 61 | 5 | MTX+LFN | 2 | 20 | 20 |  |  |  |  | 10.0 | yes |
| 67 | Infliximab | 37 | 3 | AZA+HCQ | 2 |  |  |  | 100 | 400 |  | 10.0 | yes |
| 68 | Infliximab | 52 | 3 | MTX+HCQ | 2 | 15 |  |  |  | 400 |  | 5.0 | yes |
| 69 | Infliximab | 56 | 2 | MTX+HCQ | 2 | 15 |  |  |  | 400 |  | 5.0 | yes |
| 70 | Infliximab | 71 | 3 | MTX+HCQ | 2 |  |  |  |  | 400 |  | 0.0 | yes |
| 71 | Etanercept | 71 | 4 | LFN | 1 | 15 |  |  |  |  |  | 5.0 | yes |
| 72 | Infliximab | 58 | 1 | MTX | 1 |  | 20 |  |  |  |  | 7.5 | yes |
| 73 | Infliximab | 38 | 2 | SSZ | 1 |  |  |  |  |  | 3000 | 0.0 | yes |
| 74 | Infliximab | 57 | 3 | MTX+SSZ | 2 | 20 |  |  |  |  | 2000 | 5.0 | yes |
| 75 | Etanercept | 56 | 2 | LFN+HCQ | 2 |  | 20 |  |  | 400 |  | 0.0 | yes |
| 76 | Adalimumab | 42 | 2 | MTX+HCQ | 2 | 15 |  |  |  | 400 |  | 0.0 | yes |
| 77 | Adalimumab | 56 | 2 | MTX | 1 | 20 |  |  |  |  |  | 5.0 | yes |
| 78 | Infliximab | 61 | 3 | MTX | 1 | 20 |  |  |  |  |  | 0.0 | yes |
| 79 | Infliximab | 57 | 5 | LFN+SSZ | 2 |  | 20 |  |  |  | 3000 | 7.5 | yes |
| 80 | Infliximab | 62 | 3 | MTX | 1 | 20 |  |  |  |  |  | 0.0 | yes |
| 81 | Infliximab | 56 | 4 | LFN | 1 |  | 20 |  |  |  |  | 5.0 | yes |
| 82 | Etanercept | 68 | 3 | LFN | 1 |  | 20 |  |  |  |  | 0.0 | yes |
| 83 | Etanercept | 54 | 1 | LFN+SSZ | 2 |  | 20 |  |  |  | 2000 | 0.0 | yes |
| 84 | Infliximab | 61 | 5 | CyA | 1 |  |  | 100 |  |  |  | 7.5 | yes |
| 85 | Etanercept | 72 | 2 | MTX | 1 | 20 |  |  |  |  |  | 10.0 | yes |
| 86 | Infliximab | 48 | 3 | MTX+HCQ | 2 | 20 |  |  |  | 400 |  | 0.0 | yes |
| 87 | Infliximab | 57 | 2 | LFN+HCQ | 2 |  | 20 |  |  | 400 |  | 5.0 | yes |
| 88 | Infliximab | 64 | 2 | MTX | 1 | 20 |  |  |  |  |  | 5.0 | yes |
| 89 | Infliximab | 56 | 5 | LFN | 1 |  | 20 |  |  |  |  | 7.5 | yes |
| 90 | Adalimumab | 65 | 3 | MTX+SSZ | 2 | 20 |  |  |  |  | 3000 | 7.5 | yes |
| 91 | Etanercept | 57 | 2 | MTX | 1 | 20 |  |  |  |  |  | 10.0 | yes |
| 92 | Infliximab | 58 | 3 | MTX+SSZ | 2 | 20 |  |  |  |  | 2000 | 12.5 | yes |
| 93 | Infliximab | 49 | 3 | MTX | 1 | 20 |  |  |  |  |  | 7.5 | yes |
| 94 | Etanercept | 56 | LFN | LFN | 1 |  | 20 |  |  |  |  | 5.0 | yes |
| 95 | Adalimumab | 51 | 2 | LFN | 1 |  | 20 |  |  |  |  | 5.0 | yes |

MTX: Methotrexate, LFN: leflunomide, AZA: azathioprine, CyA:cyclosporine A; HCQ: hydroxychloroquine; SSZ: sulfasalazyne; NSAID: non-steroidal anti-inflammatory drugs. * Steroid dose: mg/day prednisone
