# Supplementary material for: Circulating miRNAs as potential biomarkers of therapy effectiveness in rheumatoid arthritis patients treated with anti-TNFα
Source: Arthritis Res Ther. 2015 Mar 9;17(1):49. doi: 10.1186/s13075-015-0555-z (PMC4377058; doi:10.1186/s13075-015-0555-z)
Supplement: Additional file 2: Figure S4. — Relative miRNA levels at starting (T1) and after six months of anti-TNFα/DMARDs combination therapy (T2) in the validation cohort (n = 85). To validate the PCR array data,10 miRNAs differentially expressed were selected (hsa-miR-125b, hsa-miR-23a-3p, hsa-miR-21-5p, hsa-miR-126-3p, hsa-miR-146a-5p, hsa-let-7a-5p, hsa-miR-16-5p, hsa-miR-124a-3p, hsa-miR-155-5p, and hsa-miR-223). (A) Relative expression levels of each miRNA in the group of RA patients responders to therapy (n = 75). Boxes indicate the interval between the 25th and 75th percentiles and horizontal bars inside boxes indicate median. Whiskers indicate the interval of data within 1.5 × interquartile ranges (IQR). Closed circles indicate data points outside 1.5 × IQR. * P < 0.05. (B) Relative expression levels of each miRNA in the group of non-responders to the combination therapy (n = 10). [file 13075_2015_555_MOESM2_ESM.ppt]

## Slide 1
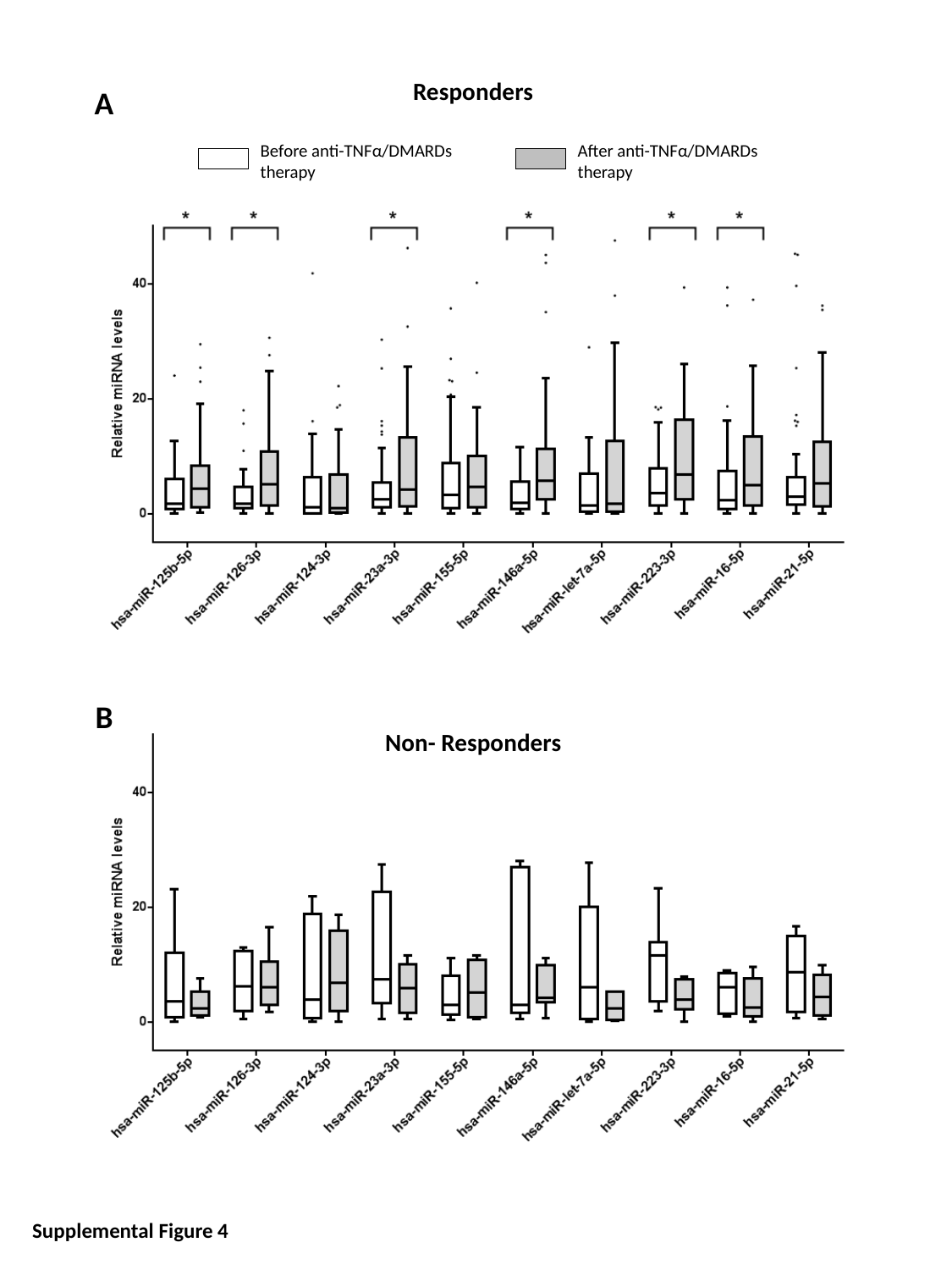

Responders
A
Before anti-TNFα/DMARDs therapy
After anti-TNFα/DMARDs therapy
B
Non- Responders
Supplemental Figure 4
