# Supplementary figures and images for: Circulating miRNAs as potential biomarkers of therapy effectiveness in rheumatoid arthritis patients treated with anti-TNFα
Source: Arthritis Res Ther. 2015 Mar 9;17(1):49. doi: 10.1186/s13075-015-0555-z (PMC4377058; doi:10.1186/s13075-015-0555-z)

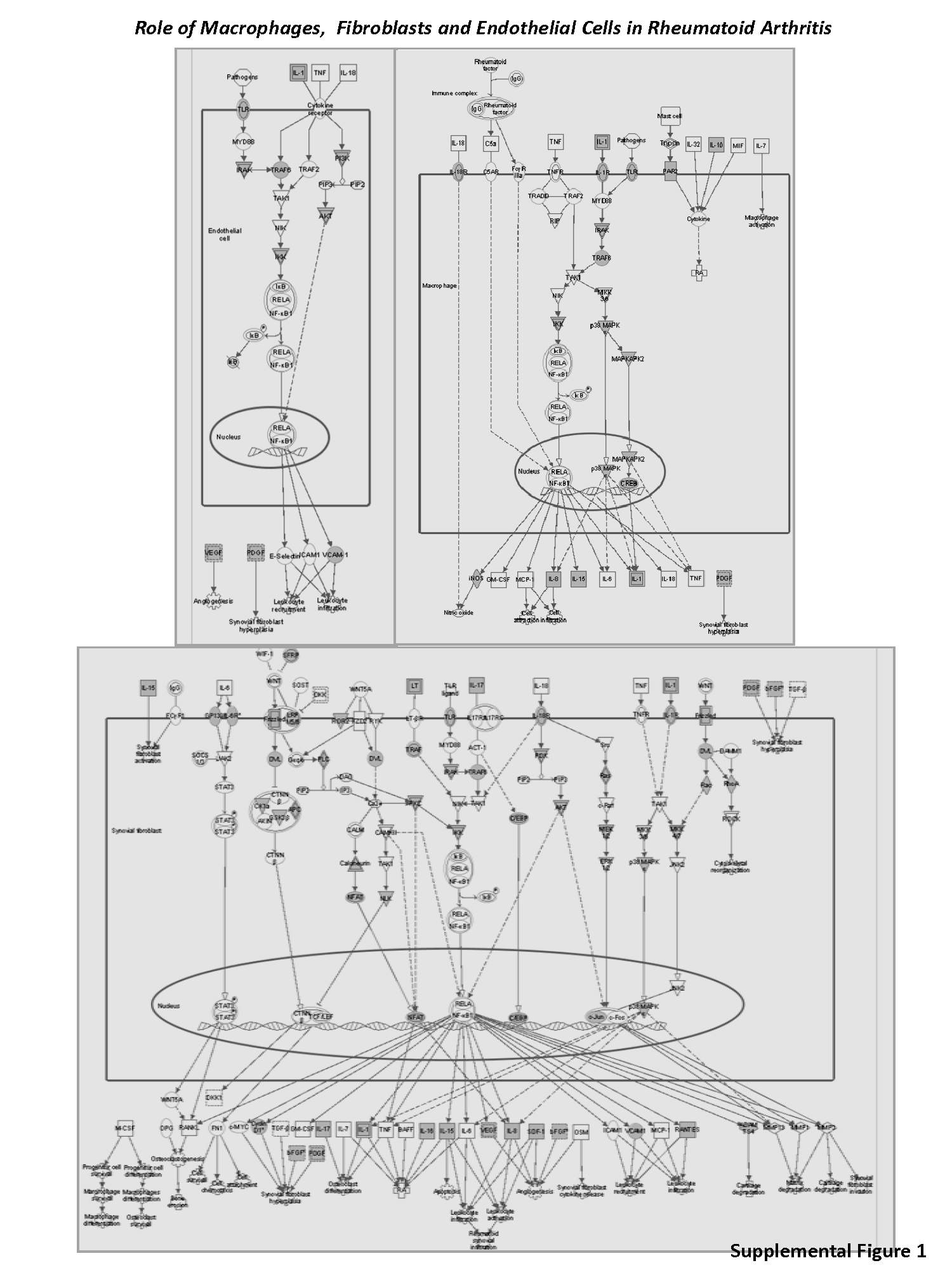

Supplement: Additional file 3: Figure S1. — Distribution of all the genes potentially modified by the validated miRNAs integrated in the pathways related to the role of macrophages, fibroblasts and endothelial cells in rheumatoid arthritis. The different points were RA-related canonical pathways might be regulated are represented by grey-filled symbols. [file 13075_2015_555_MOESM3_ESM.tiff]

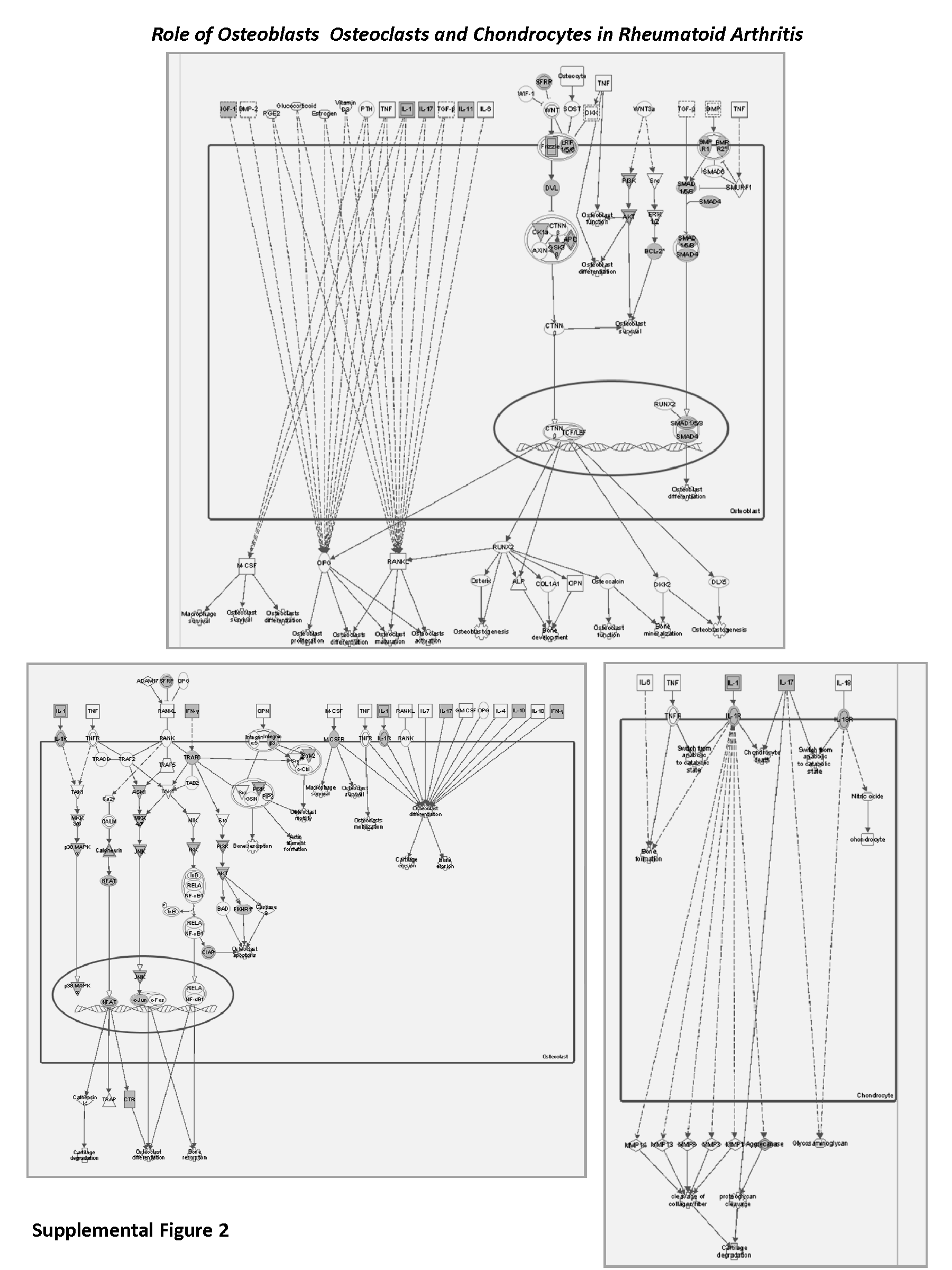

Supplement: Additional file 4: Figure S2. — Distribution of all the genes potentially modified by the validated miRNAs integrated in the pathways related to the role of osteoblasts, osteoclasts, and chondrocytes in rheumatoid arthritis. The different points were RA-related canonical pathways might be regulated are represented by grey-filled symbols. [file 13075_2015_555_MOESM4_ESM.tiff]

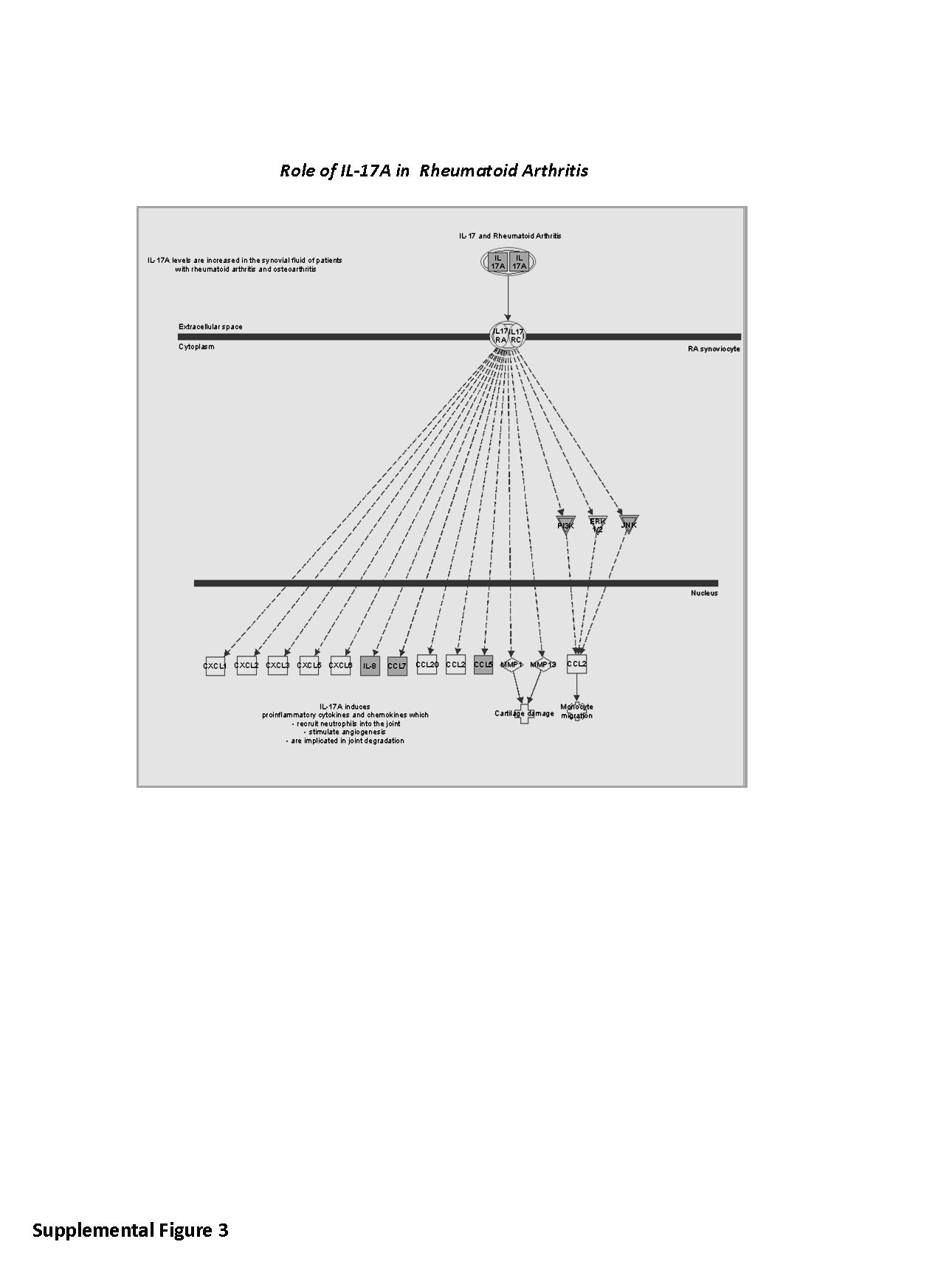

Supplement: Additional file 5: Figure S3. — Distribution of all the genes potentially modified by the validated miRNAs integrated in the pathways related to the role of IL-17A in rheumatoid arthritis. The different points were RA-related canonical pathways might be regulated are represented by grey-filled symbols. [file 13075_2015_555_MOESM5_ESM.tiff]
